# Sm complex assembly and 5′ cap trimethylation promote selective processing of snRNAs by the 3′ exonuclease TOE1

**DOI:** 10.1073/pnas.2315259121

**Published:** 2024-01-09

**Authors:** Tiantai Ma, Erica S. Xiong, Rea M. Lardelli, Jens Lykke-Andersen

**Affiliations:** ^a^Department of Molecular Biology, School of Biological Sciences, University of California, San Diego, La Jolla, CA 92093

**Keywords:** TOE1, snRNA, 3′ end processing, Sm complex, 5′ cap trimethylation

## Abstract

Competing exonucleases that promote 3′ end maturation or degradation direct quality control of small non-coding RNAs, but how these enzymes distinguish normal from aberrant RNAs is poorly understood. The Pontocerebellar Hypoplasia 7 (PCH7)–associated 3′ exonuclease TOE1 promotes maturation of canonical small nuclear RNAs (snRNAs). Here, we demonstrate that TOE1 achieves specificity toward canonical snRNAs through their Sm complex assembly and cap trimethylation, two features that distinguish snRNAs undergoing correct biogenesis from other small non-coding RNAs. Indeed, disruption of Sm complex assembly via snRNA mutations or protein depletions obstructs snRNA processing by TOE1, and in vitro snRNA processing by TOE1 is stimulated by a trimethylated cap. An unstable snRNA variant that normally fails to undergo maturation becomes fully processed by TOE1 when its degenerate Sm binding motif is converted into a canonical one. Our findings uncover the molecular basis for how TOE1 distinguishes snRNAs from other small non-coding RNAs and explain how TOE1 promotes maturation specifically of canonical snRNAs undergoing proper processing.

## Significance

RNA quality control pathways rid cells of defective RNAs by mechanisms that are often poorly understood. In the case of human small nuclear (sn)RNAs, core components of the spliceosome, previous studies identified a central role in quality control of competing 3′-to-5′ exonucleases that promote either 3′ end maturation or degradation. However, how these exonucleases distinguish functional from defective snRNAs has remained unknown. Here, we demonstrate that the 3′ end maturation exonuclease TOE1 distinguishes functional from defective snRNAs through two snRNA features, Sm-complex assembly and cap trimethylation, that signify successful snRNA biogenesis. These findings suggest that snRNA quality control relies on the specificity of TOE1 for correctly assembled snRNAs, leaving defective snRNAs to be degraded by more promiscuous decay exonucleases.

Small non-coding (nc)RNAs play key roles at all levels of gene regulation. In eukaryotes, the majority of small ncRNAs are transcribed as precursor RNAs that need to undergo further processing to become functional mature RNAs, including trimming of 3′ end extensions (1, 2). These extensions serve as central hubs for quality control, where competing 3′-to-5′ exonucleases that promote maturation or degradation dictate whether the small ncRNAs undergo processing to functional molecules (3–6) or are subjected to decay (5, 7, 8). The competition between these processes can be influenced by post-transcriptional oligo(A) or -(U) tailing by terminal nucleotidyl transferases (5, 9–12). A key question is what dictates the specificity of the exonucleases and terminal nucleotidyl transferases that act on small ncRNAs to ultimately control their fate. A prevailing hypothesis is that enzymes that promote maturation recognize specific features of RNAs undergoing proper biogenesis, while more promiscuous decay enzymes degrade those RNAs that fail to conform to canonical processing. This predicts that maturation enzymes recognize common features of canonical RNAs that signify their correct biogenesis.

Recent studies have uncovered several 3′ to 5′ exonucleases that promote deadenylation and 3′ end maturation of human small ncRNAs. Two such exonucleases are the DEDD family deadenylases target of EGR1 protein 1 (TOE1; also known as CAF1Z) (13) and poly(A)-specific ribonuclease (PARN) (14). While TOE1 and PARN are homologous proteins and are both activated by oligo(A) tails (13, 14), they differ in their specificity for small ncRNAs. PARN has been reported to process 3′ ends of several types of small ncRNAs, including small nucleolar RNAs (snoRNAs) (4, 15) and the telomerase RNA component (TERC) (16–18). By contrast, TOE1 is known to process 3′ ends of RNA Polymerase II–transcribed small nuclear RNAs (Pol II snRNAs; i.e., all snRNAs except U6 and U6atac) (3, 5), and has also been reported to process some snoRNAs, scaRNAs, and TERC (4, 19). Yet another DEDD deadenylase, the germ-cell specific PNLDC1, has been implicated in processing of Piwi-interacting (pi)RNAs (20–22), and USB1, an exonuclease of the 2H phosphodiesterase family, is known to process U6 and U6atac snRNAs and some miRNAs (23–25). Functions of these enzymes are central to human health, with TOE1 mutations associated with *Pontocerebellar Hypoplasia Type 7* (PCH7) (3), PARN mutations with *Dyskeratosis Congenita* and other human disorders (26, 27), PNLDC1 mutations with *azoospermia* (28), and USB1 mutations with *Poikiloderma with neutropenia* (29). Despite their importance in RNA metabolism and human disease, little is known about the molecular basis for the RNA specificity of these exonucleases.

SnRNAs are central components of the spliceosome that carries out pre-mRNA splicing. The maturation of Pol II snRNAs includes multiple steps in the nucleus and cytoplasm (30). During transcription, a 7-methyl guanosine (m7G) cap is co-transcriptionally added to the snRNA 5′ end and the 3′ end is cleaved by the integrator complex, leaving a short 3′ end tail (31, 32). The m7G cap subsequently promotes nuclear export via the export factor PHAX in association with the nuclear cap–binding complex (CBC) (33). In the cytoplasm, the Sm complex is loaded onto the snRNAs with the help of the SMN-Gemin2 complex, and protein arginine methyl transferases (PRMTs) (34–42), and the m7G cap is hypermethylated to a trimethylguanosine (TMG) cap by trimethylguanosine synthase 1 (TGS1) (43–45). The Sm complex and the TMG cap are subsequently recognized by Snurportin (SNUPN), which promotes nuclear import via Importin β (46, 47). Following nuclear import, the snRNAs undergo nucleotide modifications directed by scaRNAs and assemble with other snRNA-protein complexes (snRNPs) to form the spliceosome (48).

Evidence suggests that TOE1 acts on Pol II snRNAs at least twice during their maturation (5). During early biogenesis, prior to or upon nuclear export, TOE1 partially trims Pol II snRNA 3′ ends in a process that involves oligoadenylation (5). The remainder of the tail is removed later, during or after nuclear import, when TOE1 acts on snRNAs a second time (5, 49). While TOE1 acts on all canonical Pol II snRNAs, tested unstable snRNA variants transcribed from snRNA pseudogenes and mutant U1 snRNA deleted of its Sm binding site escape 3′ end processing and are instead subjected to decay by degrading exonucleases (5, 12, 49, 50). The features of canonical snRNAs recognized by TOE1 during early and late processing steps and how TOE1 distinguishes canonical snRNAs from unstable snRNA variants and other small ncRNAs of the cell has remained unknown.

In this study, we addressed how TOE1 achieves specificity toward canonical Pol II snRNAs. Global 3′ end sequencing of newly transcribed small RNAs in the absence or presence of TOE1 confirms that TOE1 specifically processes canonical Pol II snRNAs over other classes of small RNAs. Dissecting the features of Pol II snRNPs recognized by TOE1 revealed the Sm complex and the TMG cap as two key features characteristic of Pol II snRNPs that promote TOE1 processing. An unstable U1 snRNA variant known to escape TOE1-processing, U1v15, is fully processed by TOE1 when the variant Sm binding motif is converted into that of canonical snRNAs. Our findings demonstrate that TOE1-mediated snRNA maturation is driven by Sm complex assembly and cap trimethylation, features that are specific to canonical Pol II snRNAs undergoing proper biogenesis.

## Results

### TOE1 Specifically Processes Pol II snRNAs.

**Fig. 1.**
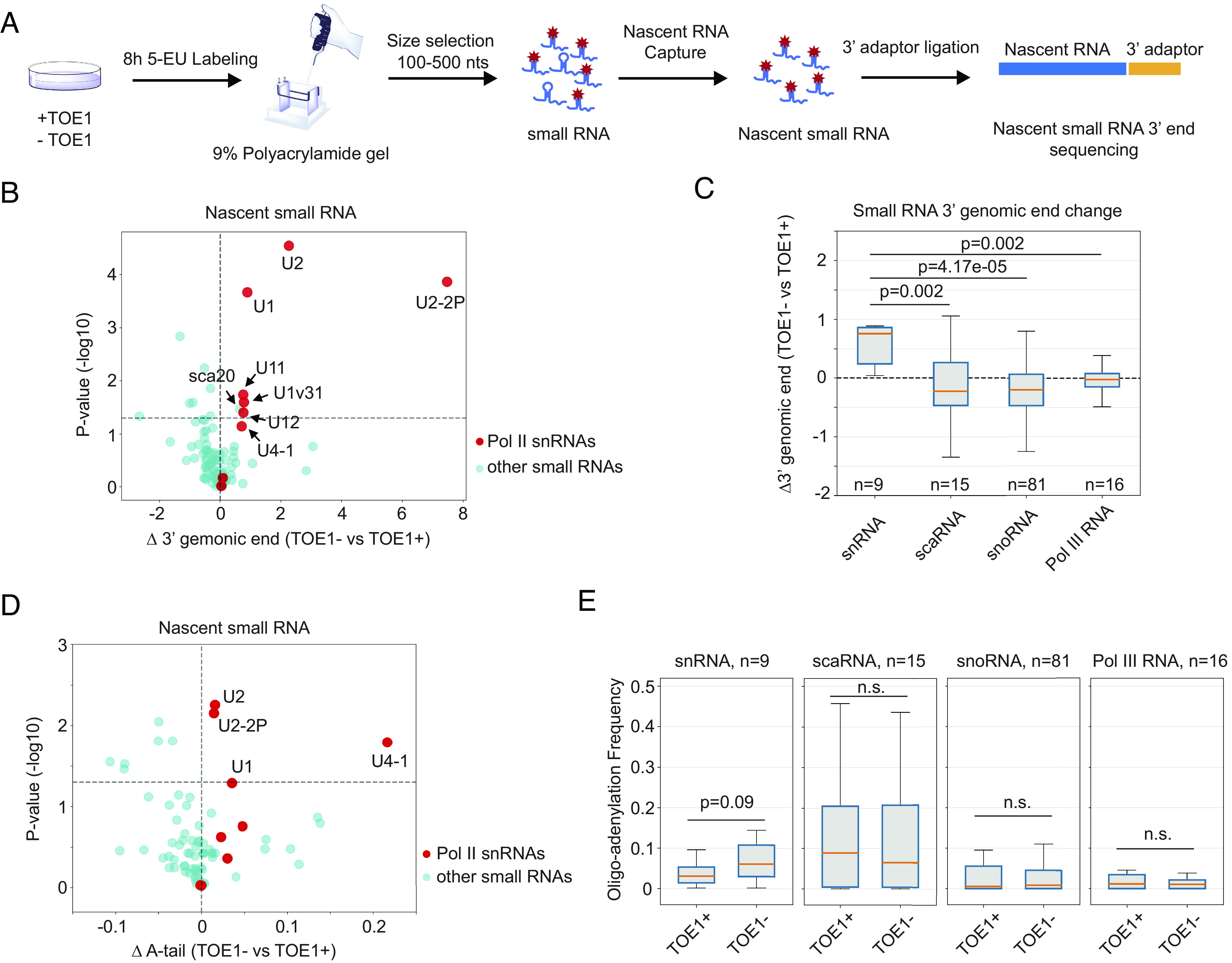
TOE1 shows specificity toward Pol II snRNAs. (*A*) Schematic of the newly transcribed small RNA global 3′ end sequencing workflow. Control (TOE1+) or TOE1 depleted (TOE1−) cells were metabolically labeled with 5-ethynyl uridine (5-EU) for 8 h followed by size selection and nascent RNA capture. Newly transcribed RNA 3′ ends were determined by global small RNA sequencing. (*B*) Scatter plot showing changes in nascent small RNA 3′ end lengths in TOE1− versus TOE1+ cells and corresponding *P*-values. Each dot represents an individual small ncRNA with Pol II snRNAs in red and other small ncRNAs in cyan. *P*-values were calculated from three individual biological repeats by Student’s two-tailed *t*-test and plotted on a −log10 scale. The horizontal dashed line represents *P* = 0.05. Small ncRNAs on the *Right* side of the plot have extended 3′ end tails upon TOE1 depletion. (*C*) Box and whisker plots showing changes in 3′ end lengths of groups of related small ncRNAs in TOE1− versus TOE1+ cells. The number (*n*) of RNAs in each group is indicated. Boxes represent quartiles around median values shown as orange lines, with whiskers extending to maximum and minimum values within 1.5 times the interquartile range from the boxes. *P*-values were calculated by comparing each group of small ncRNAs to the group of Pol II snRNAs using the two-sample Kolmogorov–Smirnov test. (*D*) Scatter plot of changes in frequencies of nascent small RNAs containing 3′ end posttranscriptional oligo(A) tails, defined as two or more post-transcriptional adenosines, in TOE1− versus TOE1+ cells. Dots and axes are labeled as in panel *B*. (*E*) Box and whisker plots showing distributions of 3′ end oligoadenylation (two or more adenosines) frequencies for each group of small ncRNAs in TOE1+ or TOE1− cells. Shown *P*-values were calculated using Student’s two-tailed *t*-test comparing TOE1− to TOE1+ conditions.

RNAs affected at steady state by TOE1 depletion have been previously globally monitored (4). Since the processing of stable RNAs is better captured in the newly transcribed RNA population, to further delineate the repertoire of small RNA targets of TOE1, we isolated newly transcribed small RNAs, 100 to 500 nucleotides in length, from human embryonic kidney 293 T (HEK293T) cells under normal or TOE1-depleted conditions and subjected them to global 3′ end sequencing (Fig. 1*A* and *SI Appendix*, Fig. S1 *A* and *B*) (51). Comparing mean genomic-encoded tail lengths of small RNAs in the absence or presence of TOE1 revealed Pol II snRNAs as the primary targets of TOE1-mediated 3′ end processing (Fig. 1 *B* and *C*). A subset of Pol II snRNAs also accumulated short oligo(A) tails in the absence of TOE1 (Fig. 1 *D* and *E*). Only one small ncRNA that was not an snRNA, scaRNA20, was observed to be significantly extended in the absence of TOE1 (Fig. 1*B* and *SI Appendix*, Fig. S1*C*), primarily due to an extended oligo(A) tail (*SI Appendix*, Fig. S1*D*). While our global sequencing assays captured the majority of Pol II snRNAs as targets of TOE1, U5 and U4atac snRNAs were of too low abundance in the sequencing assays to include in the analysis; however, these have both previously been identified as TOE1 targets in gene-specific sequencing experiments (3, 5). Collectively, our global 3′ end sequencing analyses of newly transcribed small RNAs show TOE1 specificity toward Pol II snRNAs as a class, although TOE1 may also act on a subset of other small ncRNAs, including scaRNA20, consistent with previous reports (4, 19). This raised the question of how TOE1 achieves specificity for Pol II snRNAs over other small ncRNAs.

### Mutations in Sm Complex and U1-70 K Binding Motifs of U1 snRNA Impair TOE1-Mediated 3′ End Processing.

**Fig. 2.**
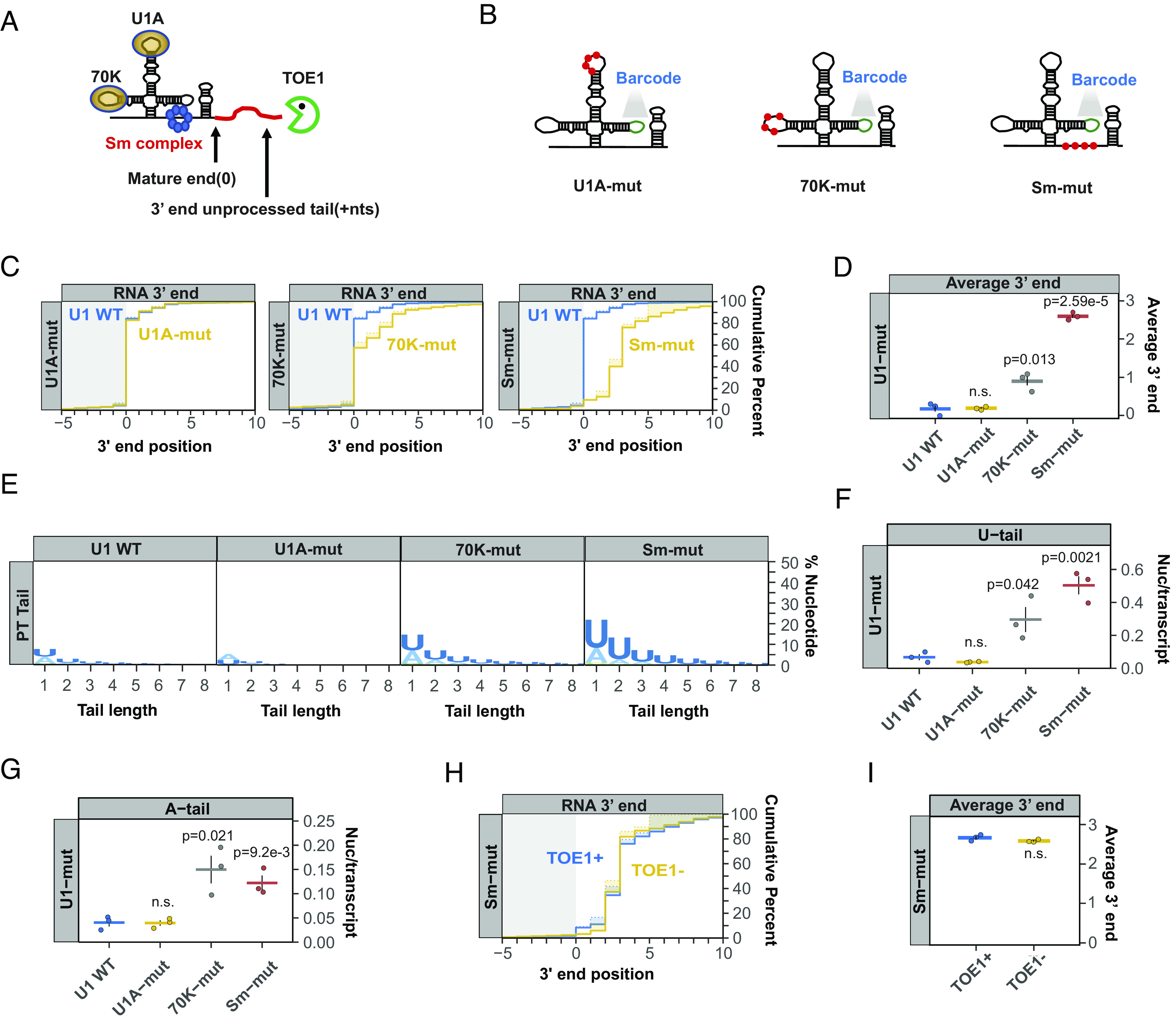
Mutations in Sm complex– and U1-70 K-binding motifs of U1 snRNA inhibit 3′ end processing. (*A*) Schematic of the U1 snRNP with 70 K, U1A, and Sm complex components shown at their binding motifs and a red line representing a 3′ end unprocessed tail. The snRNP schematic is not proportional to size. A proportional U1 snRNA-Sm complex model (54) can be found in *SI Appendix*, Fig. S2*A*. (*B*) Schematics of barcoded U1 snRNAs with mutations known to abolish binding of U1A (U1A-mut), U1-70 K (70 K-mut) or the Sm complex (Sm-mut) (*SI Appendix*, Table S2) shown as red dots. A barcode sequence in stem loop 3 is indicted in green. (*C*) Cumulative plots showing distributions of 3′ end positions for each U1 snRNA mutant (yellow) as compared to wild-type U1 snRNA (U1 WT, blue) as determined by U1 snRNA gene-specific 3′ end sequencing of steady-state exogenous snRNAs. Mature 3′ ends are represented as position 0 with positive numbers representing unprocessed 3′ end tails. Solid lines represent actual snRNA 3′ end positions, while dotted lines represent genome-encoded 3′ end positions with shading in between representing post-transcriptionally added nucleotides. Only RNAs terminating at or downstream of the −10 position were analyzed. Three individual biological repeats were averaged for each plot. (*D*) Average 3′ end positions for each U1 WT or mutant snRNAs with each dot representing an individual biological repeat. Black vertical lines represent SEM and *P*-values were calculated using Student’s two-tailed *t*-test comparing U1 mutants to U1 WT (*P* > 0.05 is noted as n.s.). (*E*) Sequence logo plots representing percentages of post-transcriptional added nucleotides for U1 WT and U1 mutant snRNAs. (*F* and *G*) Average post-transcriptional added uridines (*F*) and adenosines (*G*) per transcript for U1 WT and U1 mutants plotted as in panel *D*. (*H*) Cumulative plot showing 3′ end distributions for the Sm mutant U1 snRNA under TOE1+ (blue) or TOE1− (yellow) conditions. (*I*) Average 3′ end positions for the Sm mutant U1 snRNA in TOE1+ (blue) and TOE1− (yellow) conditions.

To investigate how TOE1 achieves substrate specificity, we turned to U1 snRNA (Fig. 2*A* and *SI Appendix*, Fig. S2*A*). Reasoning that TOE1 may recognize specific protein components of the U1 snRNP, we introduced previously described mutations that disrupt the interaction of U1 snRNA with U1A, U1-70 K, and the Sm complex (Fig. 2*B*) (40, 52, 53). These mutations were introduced into a bar-coded exogenous U1 snRNA (3, 50), which allowed us to monitor their effect on U1 snRNA processing by 3′ end sequencing. Consistent with early observations in Xenopus oocytes (49), disruption of the Sm complex binding motif led to a strong defect in U1 snRNA 3′ end processing (Fig. 2 *C* and *D*). In addition, mutating the U1-70 K binding motif caused a minor, but statistically significant, defect in U1 snRNA 3′ end processing, whereas disrupting the U1A binding motif did not impair processing (Fig. 2 *C* and *D*). Further analyses of the mutant U1 snRNA 3′ ends revealed that the disruption of the Sm complex binding motif caused a significant fraction of the U1 snRNA population to accumulate with oligo(U) tails (Fig. 2 *E* and *F*), consistent with recent observations (12). Interestingly, U1 snRNA mutated for U1-70 K binding also accumulated oligo(U) tails, and both Sm and U1-70 K binding mutants showed oligo(A) tailing as well (Fig. 2 *E* and *G*). Monitoring 3′ end processing of the mutant U1 snRNAs in the presence or absence of TOE1 depletion revealed that disruption of the Sm complex binding motif fully abolished the ability of TOE1 to process U1 snRNA (Fig. 2 *H* and *I*), whereas processing by TOE1 is only partially defective for the U1-70 K binding mutant (*SI Appendix*, Fig. S2 *B* and *C*).

### Sm Complex Depletion Impairs 3′ End Processing of Multiple Pol II snRNAs.

**Fig. 3.**
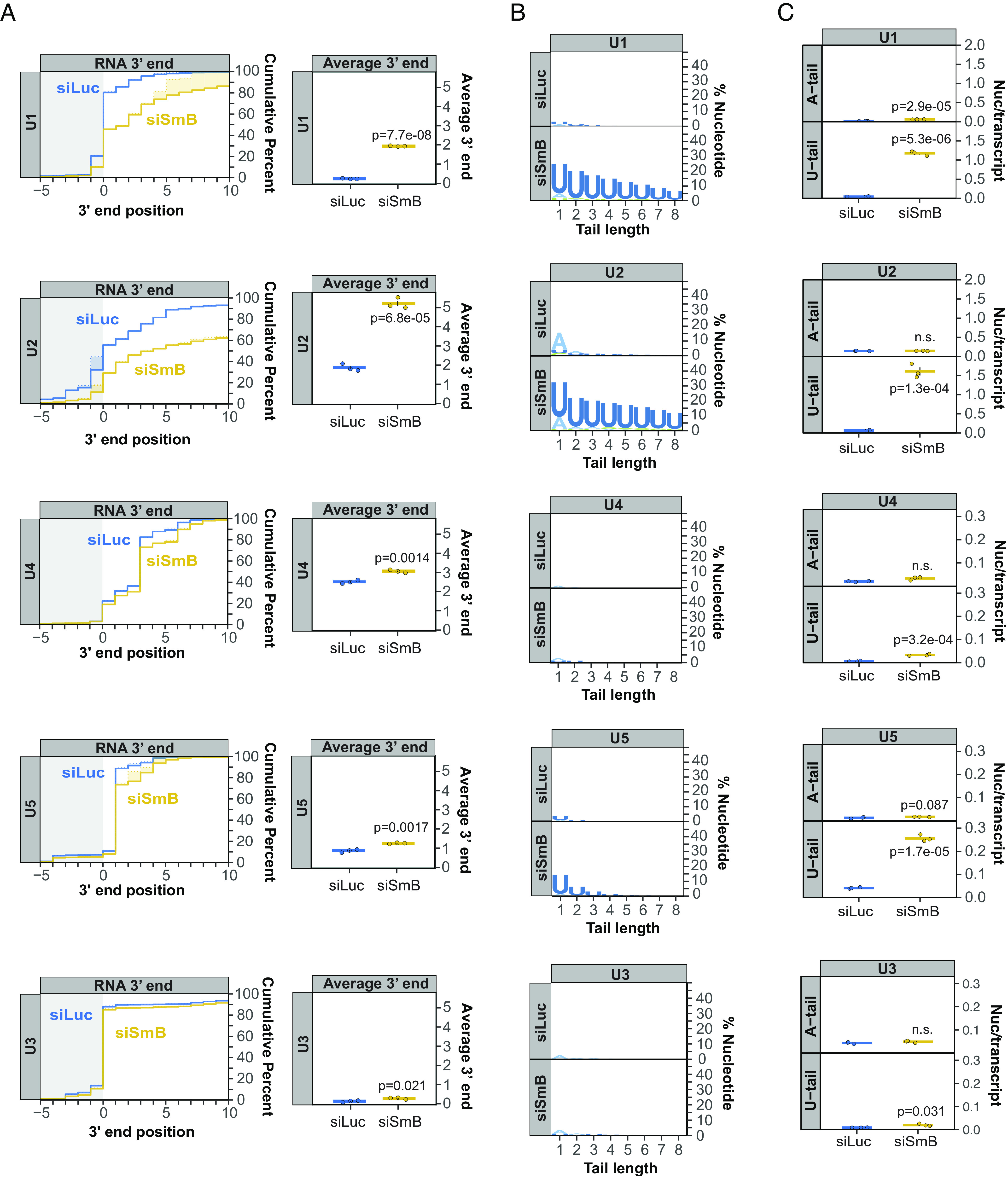
Sm complex depletion impairs 3′ end processing of multiple Pol II snRNAs. (*A*) Cumulative plots showing 3′ end distributions of newly transcribed U1, U2, U4, and U5 snRNAs in control (siLuc; blue) versus SmB (siSmB; yellow) knock-down conditions. U3 snoRNA serves as a negative control. The average of three individual biological repeats is plotted and average 3′ end positions are graphed on the *Right* with dots representing individual biological repeats and SEMs shown as vertical black lines. *P*-values were calculated by Student’s two-tailed *t*-test. (*B*) Sequence logo plots representing the percentage of post-transcriptionally added nucleotides for snRNAs and U3 snoRNA under control (siLuc) or SmB knock-down (siSmB) conditions. (*C*) Average number of post-transcriptionally added adenosine or uridine nucleotides per transcript for major class snRNAs and U3 snoRNA plotted as in panel *A*.

The experiments above were performed using exogenous bar-coded U1 snRNAs. To verify the importance of snRNP proteins in snRNA processing, we next monitored effects of their depletion on processing of endogenous snRNAs. Sm complex assembly is a feature characteristic of all Pol II snRNAs. To test whether Sm complex assembly is necessary for 3′ end processing of Pol II snRNAs in general, we depleted the Sm complex component SmB (*SI Appendix*, Fig. S3*A*) and performed targeted 3′ end sequencing of newly transcribed Pol II snRNAs. All tested Pol II snRNAs showed defects in 3′ end processing following SmB depletion as compared to the negative control U3 snoRNA, although some snRNAs were affected more than others (Fig. 3*A* and *SI Appendix*, Fig. S3). U1 and U2 snRNAs showed strong defects in 3′ end processing, whereas U4, U5, and U4atac snRNAs showed significant, but less extensive, defects. A majority of the Pol II snRNAs also accumulated oligo(U) tails to various degrees upon SmB depletion (Fig. 3 *B* and *C*). These tails were most prominent for U1, U2, and U5 snRNAs and observed at much lower levels for U4 and U4atac snRNAs (Fig. 3 *B* and *C* and *SI Appendix*, Fig. S3). The control U3 snoRNA showed a very minor increase in uridylation upon SmB depletion (Fig. 3 *B* and *C*). Collectively, our findings demonstrate a general role for the Sm complex in 3′ end processing of Pol II snRNAs, although some snRNAs are more sensitive to Sm complex levels than others.

### U1-70 K Compensates for a Suboptimal Sm Binding Motif to Stimulate U1 snRNA 3′ End Processing.

**Fig. 4.**
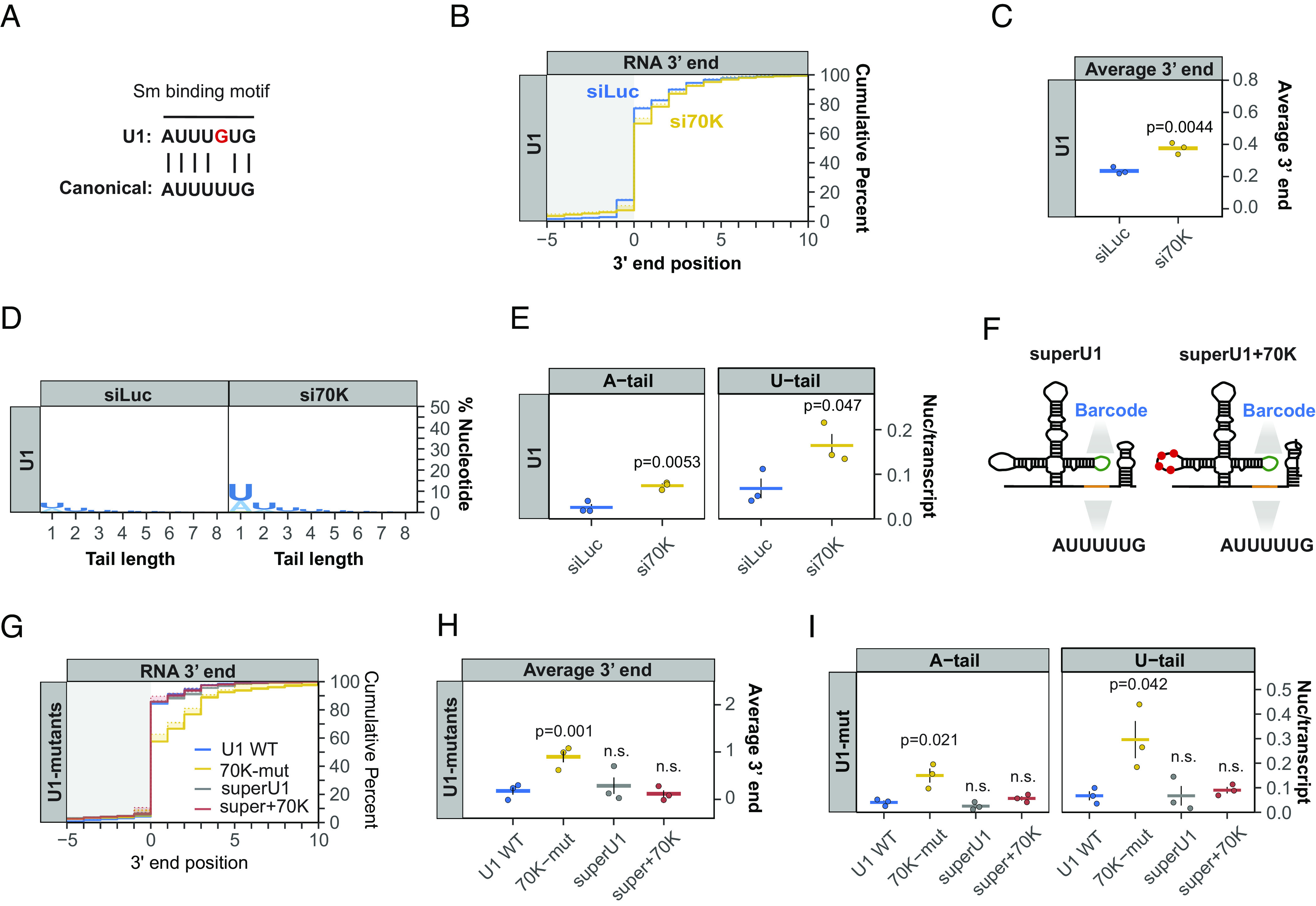
U1-70 K compensates for a suboptimal Sm binding motif to stimulate U1 snRNA 3′ end processing. (*A*) Comparison of the U1 snRNA Sm binding motif with the canonical Sm binding motifs found in other Pol II snRNAs. (*B*) Cumulative plot showing the 3′ end distributions of newly transcribed U1 snRNA in control (siLuc, blue) or U1-70 K depletion (si70K, yellow) conditions. Averages of three individual biological repeats are plotted. (*C*) Average newly transcribed U1 snRNA 3′ end positions from the experiments plotted in panel *B* with dots representing individual biological repeats and SEMs shown as vertical black lines. The *P*-value was calculated by Student’s two-tailed *t*-test. (*D*) Sequence logo plot representing the percentage of post-transcriptionally added nucleotides in control (siLuc) or U1-70 K depleted (si70K) conditions. (*E*) Average post-transcriptional added adenosines and uridines per transcript plotted as in panel *C*. (*F*) Schematics of barcoded exogenous U1 snRNAs with canonical Sm binding motif (superU1) and superU1 with mutations that abolish U1-70 K binding shown as red dots (superU1+70 K). (*G*) Cumulative plot showing 3′ end distributions for indicated exogenous U1 snRNAs at steady state. (*H*) Average 3′ end positions for indicated exogenous U1 snRNAs, plotted as in panel *C*. (*I*) Average post-transcriptionally added adenosine and uridine nucleotides per transcript for indicated exogenous U1 snRNAs, plotted as in panel *E*.

In contrast to the Sm complex, which assembles with all Pol II snRNAs, U1-70 K is unique to U1 snRNA. Previous studies have shown that U1-70 K helps promote assembly of the Sm complex onto U1 snRNA, which contains a suboptimal Sm binding site (34) (Fig. 4*A*). Consistent with the effect of the U1-70 K binding-site mutation, depletion of the U1-70 K protein impaired 3′ end processing of newly transcribed U1 snRNA and caused low-level oligo(A) and oligo(U) tailing (Fig. 4 *B*–*E* and *SI Appendix*, Fig. S4*A*). To test whether U1-70 K promotes U1 snRNA 3′ end processing via its stimulation of Sm complex assembly, we tested the effect of converting the suboptimal U1 snRNA Sm binding motif into a canonical one (Fig. 4*F*, superU1), which is known to promote assembly of the Sm complex independently of U1-70 K (34). The canonical Sm binding site rescued the 3′ end processing defect of the U1-70 K binding site mutation and strongly reduced adenylation and uridylation (Fig. 4 *G*–*I*). Thus, U1-70 K promotes U1 snRNA 3′ end processing by compensating for a suboptimal Sm complex binding site. In contrast to U1-70 K and the Sm complex, depletion of U1 snRNP components U1A and U1C showed no defect in U1 snRNA 3′ end processing (*SI Appendix*, Fig. S4 *B*–*G*), consistent with our observations with the U1A-binding mutant U1 snRNA (Fig. 2).

### The U1 snRNA Variant U1v15 Escapes TOE1 Processing due to Variant Nucleotides in Its Sm-Binding Motif.

**Fig. 5.**
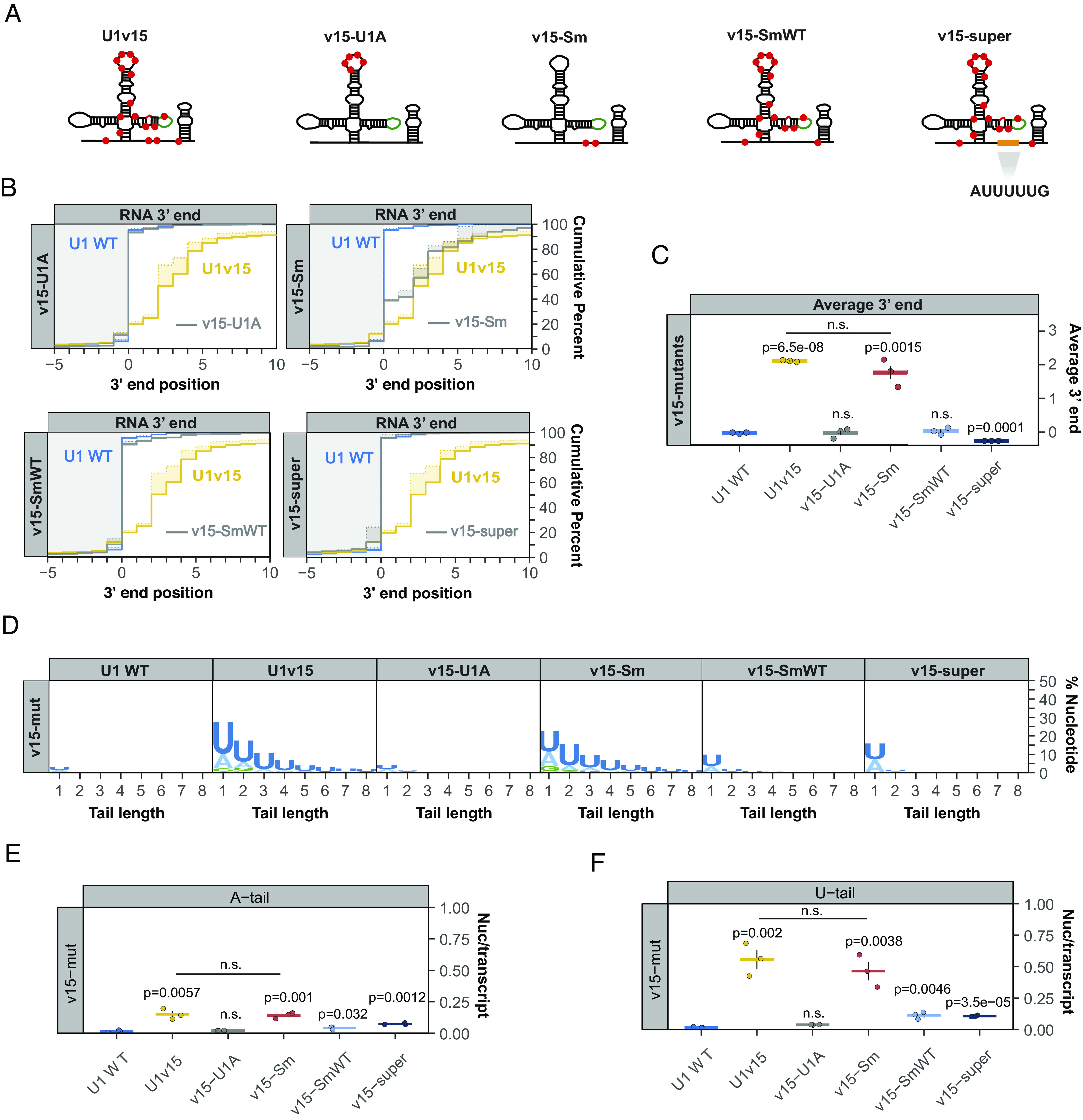
U1 snRNA variant U1v15 escapes TOE1 processing due to variant nucleotides in its Sm binding motif. (*A*) Schematic of U1 snRNA variant U1v15 and U1 snRNA mutants (v15-U1A, v15-Sm, v15-SmWT) (*SI Appendix*, Table S2) containing mutations corresponding to U1v15 nucleotide variations indicated by red dots. v15-super corresponds to U1v15 with a canonical Sm binding motif. (*B*) Cumulative plots showing 3′ end distributions for U1 WT (blue), U1v15 (yellow), and each v15 mutant (gray) at steady state. The average of three individual biological repeats is plotted. (*C*) Average 3′ end positions for U1 WT, U1v15, and v15 mutants. Dots represent individual repeats and vertical lines are SEM. *P*-values were calculated by Student’s two-tailed *t*-test by comparing v15 mutants to U1 WT. (*D*) Sequence logo plots representing the percentage of posttranscriptional added nucleotides for U1 WT, U1v15, and v15 mutants. (*E* and *F*) Average post-transcriptionally added adenosines (*E*) and uridines (*F*) per transcript plotted as in panel *C*.

The human U1 snRNA variant U1v15 is known to evade TOE1 recognition and undergo rapid degradation (5). To identify the nucleotide variations within U1v15 RNA that inhibit processing by TOE1, we introduced variant nucleotides found within the U1A or Sm binding motifs of U1v15 into the bar-coded canonical U1 snRNA (Fig. 5*A*). The variant nucleotides of the U1v15 U1A binding motif showed no effect on 3′ end processing when introduced into the canonical U1 snRNA (Fig. 5 *B* and *C*). By contrast, the variant nucleotides of the Sm binding motif were sufficient to trigger a 3′ end processing defect similar to that seen for the variant U1v15 snRNA (Fig. 5 *B* and *C*), and promote similar levels of oligo(U) and -(A) tailing (Fig. 5 *D*–*F*). We next converted the variant Sm binding site of U1v15 RNA into the Sm binding motif found in U1 snRNA (Fig. 5*A*, v15-SmWT) or the canonical Sm binding motif (Fig. 5*A*, v15-super), while leaving all other variant nucleotides intact. These modifications rescued 3′ end processing of the U1v15 snRNA variant (Fig. 5 *B* and *C*), although a minor residual level of mono-adenylation and -uridylation could be observed (Fig. 5 *D–F*). Taken together, these observations show that an intact Sm complex binding site is not only necessary for the processing of canonical Pol II snRNAs by TOE1, but can also rescue the processing of a normally unprocessed and unstable snRNA variant.

### TOE1 Directly Recognizes the Sm Complex–Assembled U1 snRNP.

**Fig. 6.**
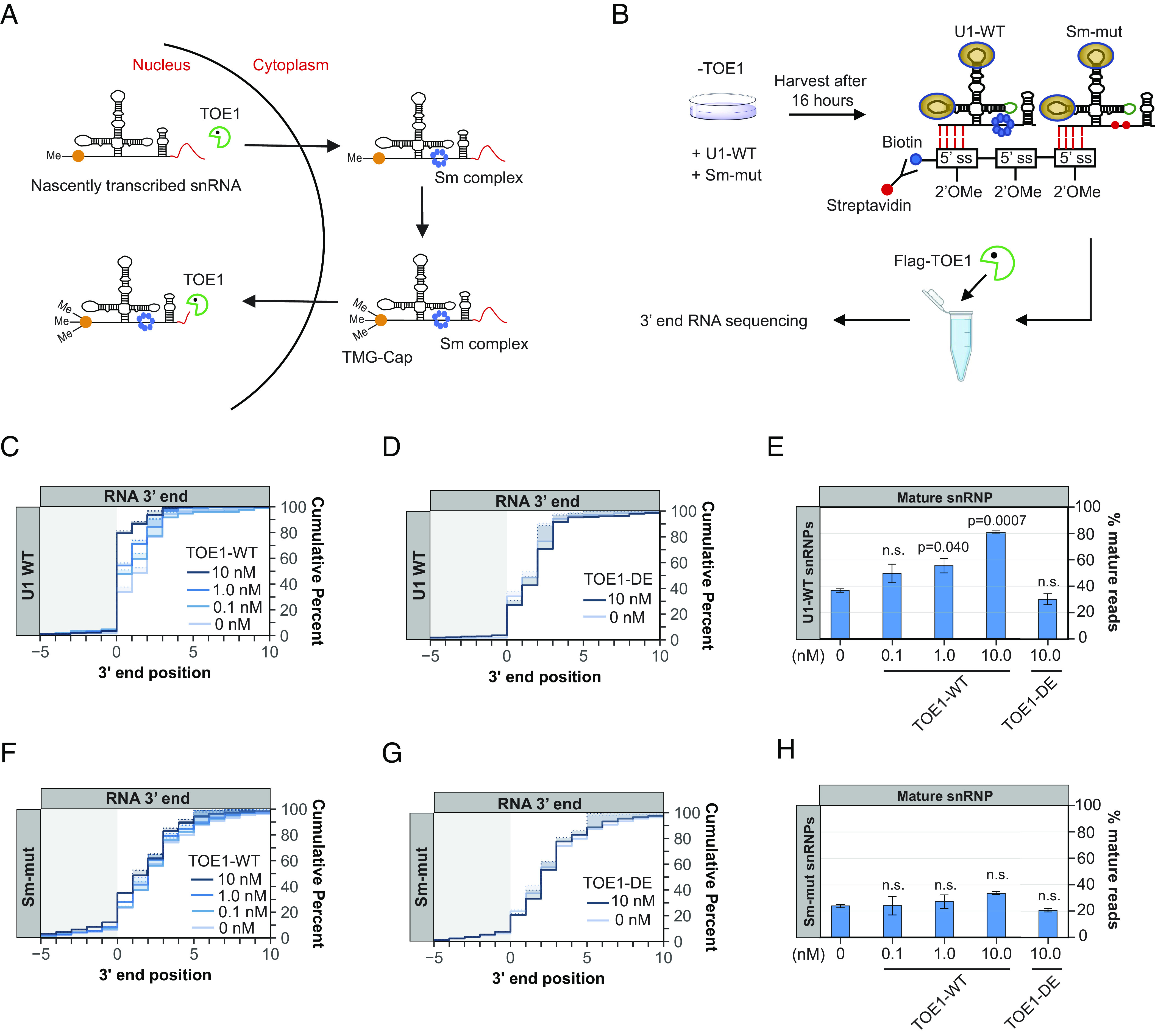
TOE1 directly recognizes the Sm complex–assembled U1 snRNP. (*A*) Schematic of the Pol II snRNA biogenesis pathway Sm complex assembly, 5′ cap trimethylation, and TOE1-mediated processing steps. The red lines at the 3′ end represent 3′ end extensions. snRNP models are not proportional to actual size. (*B*) Schematic of the in vitro U1 snRNP pull-down and TOE1 processing assay. See *Materials and Methods* for details. (*C* and *D*) Cumulative plots showing 3′ end distributions of wild-type U1 (U1 WT) snRNPs after incubation with TOE1 (panel *C*) or catalytically inactive TOE1 (TOE1 DE, panel *D*) at indicated concentrations. The average of two individual biological repeats is plotted. (*E*) Percentage of fully processed U1 WT snRNPs after TOE1 or TOE1 DE incubation at indicated concentrations. *P*-values were calculated by Student’s one-tailed *t*-test by comparing with the 0 nM TOE1 condition (n.s.: *P* > 0.05). (*F*–*H*) Same as panels *C–E* but analyzing the U1 Sm-mut snRNP.

The assembly of the Sm complex onto Pol II snRNAs takes place in the cytoplasm and triggers subsequent 5′ cap trimethylation and nuclear import (43–47) (Fig. 6*A*). Since TOE1 is known to concentrate in the nucleus (4, 13), our findings raised the question of whether TOE1 directly recognizes the Sm complex–assembled snRNP. Alternatively, the Sm complex could promote TOE1-mediated processing by indirect means such as via snRNA nuclear import. To address this question, we tested the ability of recombinant TOE1 to process wild-type or Sm-mutant snRNPs isolated from cells. A 2′-OMe-RNA oligonucleotide (*SI Appendix*, Table S1) hybridizing to the U1 snRNA 5′ splice site recognition motif was used to isolate bar-coded U1 wild-type or Sm-mutant snRNP complexes exogenously expressed in TOE1-depleted HEK293T cells (Fig. 6*B* and *SI Appendix*, Fig. S5). Isolated snRNPs were subsequently incubated with Flag-tagged TOE1 protein in vitro and analyzed by 3′ end sequencing. We found that wild-type U1 snRNP was increasingly processed at the 3′ end with increasing concentrations of TOE1, but not with a previously characterized catalytically inactive TOE1 mutant, TOE1-DE (3) (Fig. 6 *C*–*E*). By contrast, the Sm-mutant U1 snRNP showed little processing by TOE1 (Fig. 6 *F*–*H*). These observations demonstrate that TOE1 directly recognizes the Sm complex–assembled U1 snRNP. This could occur either via direct interaction with the Sm complex or by recognition of snRNP modifications downstream of Sm complex assembly.

### snRNA Processing by TOE1 Is Activated by the 5′ TMG Cap.

**Fig. 7.**
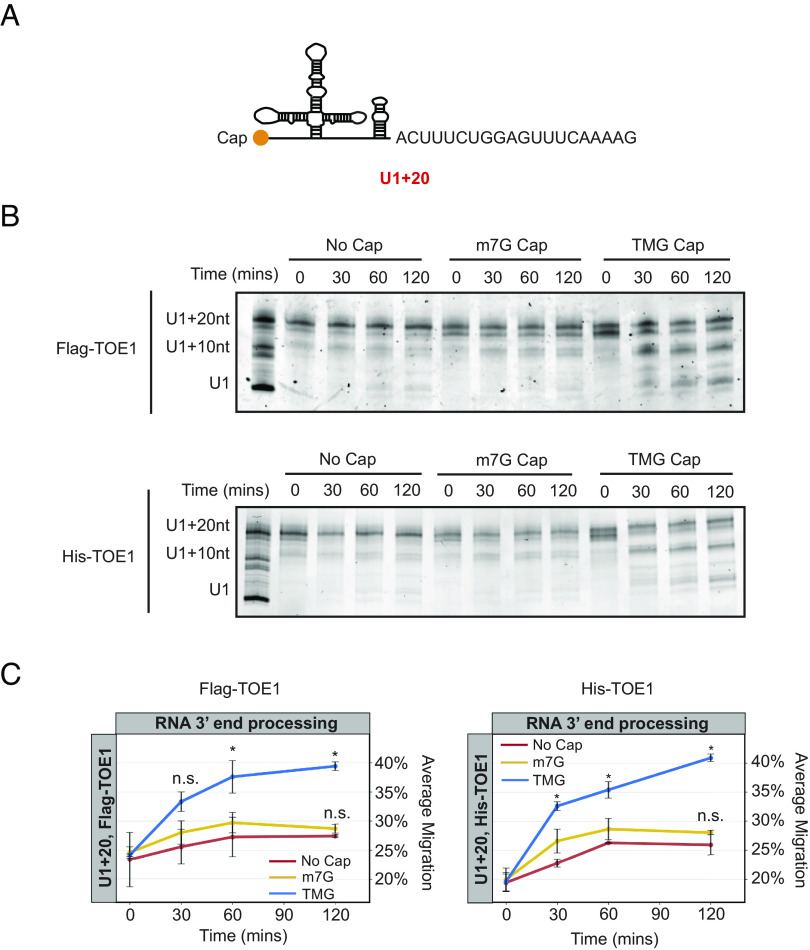
snRNA processing by TOE1 is stimulated by the 5′ TMG cap. (*A*) Schematic of in vitro transcribed U1 snRNA with a 20-nucleotide (nt) genomic-encoded tail (U1+20). (*B*) Representative denaturing gels showing uncapped, m7G-, or TMG-capped U1+20 incubated with Flag-TOE1 or His-TOE1 in vitro for indicated times. The RNA ladder consists of U1 snRNA with no tail, a 10-nt, or a 20-nt tail. (*C*) Quantification of Flag-TOE1 and His-TOE1 processing in panel *B*. The average RNA migration for each condition was quantified by ImageJ (with the U1+20-nt marker corresponding to 0% migration and U1 corresponding to 100% migration). The average of two individual biological repeats is plotted. *P*-values were calculated by Student’s two-tailed *t*-test comparing m7G or TMG U1+20 migration to the uncapped U1+20 migration at each timepoint (*: *P* < 0.05; n.s.: *P* > 0.1).

In addition to promoting nuclear import, Sm complex assembly also stimulates trimethylation of the snRNA cap (43–45). Interestingly, the TOE1 homolog PARN is known to be activated by an m7G-monomethyl cap (55–57). To test whether TOE1 activity is affected by the snRNA cap structure, we generated uncapped, m7G-capped, or TMG-capped U1 snRNAs by in vitro transcription (*SI Appendix*, Fig. S6 *A–C*). Each U1 snRNA contained a 20-nucleotide genomic-encoded tail to allow for monitoring of TOE1 activity (U1+20, Fig. 7*A*). Incubating each of the differently capped U1 snRNAs with Flag-tagged TOE1 isolated after overexpression in HEK293T cells (*SI Appendix*, Fig. S6*D*), we found that TOE1 processed the 5′ TMG-capped U1 snRNA much more efficiently than the corresponding 5′ uncapped or m7G-capped U1 snRNA substrates (Fig. 7 *B* and *C*). This effect was independent of co-purified proteins from human cells, as His-tagged TOE1 isolated from *E. coli* was similarly stimulated by the TMG cap (Fig. 7 *B* and *C* and *SI Appendix*, Fig. S6*D*). The TMG cap analog used in this assay produces an RNA population of which at most half are TMG capped (*SI Appendix*, Fig. S6 *A* and *B*), which may explain why about half of the RNA population remains unprocessed by TOE1 (Fig. 7*B*). Collectively, our observations demonstrate that TOE1 achieves substrate specificity through Sm complex assembly and cap trimethylation, two features that distinguish Pol II snRNAs undergoing proper assembly from other small ncRNAs of the cell.

## Discussion

Pontocerebellar Hypoplasia 7–associated protein TOE1 is a key 3′ end maturation factor for canonical Pol II snRNAs, but how it distinguishes substrate from non-substrate RNAs has remained unknown. Here, we present evidence that TOE1 selectively processes Pol II snRNAs over other classes of small non-coding RNAs (Fig. 1). This specificity is imparted in part by Sm complex assembly, a distinguishing feature of Pol II snRNAs. Indeed, manipulations known to inhibit Sm complex assembly, including U1 snRNA mutations and nucleotide variations (Figs. 2 and 5), and depletion of an Sm complex component or U1-70 K (Figs. 3 and 4), impair TOE1-mediated snRNA 3′ end processing. TOE1 directly recognizes the Sm complex–assembled snRNP as demonstrated by its specificity toward wild-type over Sm-mutant U1 snRNP in an in vitro 3′ end processing assay (Fig. 6). This recognition could occur via direct interaction with the Sm complex and/or with a downstream Sm complex–dependent modification of the snRNP. Indeed, TOE1 directly recognizes the trimethylated snRNA cap as evidenced by the specificity of TOE1 for a 5′ TMG capped snRNA in vitro (Fig. 7). These findings reveal the molecular basis for how TOE1 distinguishes canonical Pol-II snRNAs from other small ncRNAs of the cell.

When and where during their maturation does TOE1 process snRNAs? The biogenesis of Pol II snRNAs involves both nuclear and cytoplasmic processes (Fig. 6*A*). We previously presented evidence that TOE1 acts on snRNAs at least twice during their biogenesis, by first partially processing snRNAs prior to or during nuclear export, before completing maturation during or after nuclear re-import (5). Our findings in this study provide a molecular basis for how TOE1 achieves specificity toward snRNAs during late-stage biogenesis. At this point, snRNAs have undergone Sm complex assembly and cap trimethylation, the two features shown here to promote processing by TOE1. Since snRNA cap trimethylation occurs as a consequence of Sm complex assembly (43–45), it is possible that this late-stage processing by TOE1 occurs solely through TOE1 recognition of the trimethylated cap. Consistent with a key role for the TMG cap, depletion of TGS1, the enzyme that carries out cap trimethylation, has been reported to lead to accumulation of 3′ end extended snRNAs (58, 59). It remains to be determined how the TMG cap stimulates TOE1 activity. We observed no evidence for allosteric activation of TOE1 on an uncapped U1 snRNA by a TMG cap analog added in trans (*SI Appendix*, Fig. S7*A*). In addition to recognizing the TMG cap, TOE1 may also directly recognize the Sm complex. Consistent with this additional complexity in snRNP recognition, depletion of the Sm complex component SmB differentially affected the processing of different snRNAs by TOE1 (Fig. 3), but we observed no correlation with the extent to which cap trimethylation was affected (*SI Appendix*, Fig. S7*B*). The early-stage processing of snRNAs by TOE1, which occurs prior to Sm complex assembly and cap trimethylation, is likely a consequence of the activity of TOE1 as a deadenylase (3–5, 13). Consistent with this idea, TOE1 can process a U1 snRNA containing an oligo(A)-tail to maturity in vitro, independent of its cap structure (*SI Appendix*, Fig. S7 *C*-*E*).

Hundreds of unstable snRNA variants are encoded in the human genome (60, 61). We previously observed that TOE1 distinguishes canonical snRNAs from tested unstable snRNA variants, leaving the latter unprocessed (5). Our observations in this study demonstrate that U1v15 RNA escapes TOE1 recognition specifically due to nucleotide variations in the Sm binding motif (Figure 5). These same nucleotide variations also trigger 3′ end oligo(U) and -(A) tailing (Fig. 5), features known to promote degradation by exonucleases and the decapping machinery (10–12, 50). Thus, multiple layers of quality control act on U1v15 RNA to prevent it from assembling into spliceosomes. This likely represents general mechanisms that serve to repress the subset of transcribed snRNA variants that have acquired nucleotide variations that impair Sm complex assembly, either in the Sm binding motif or in other regions of the snRNA that affect Sm binding, such as the U1-70 K binding site (Figs. 2 and 4).

A large number of small non-coding RNAs are processed by enzymes of the DEDD deadenylase family, including TOE1 and PARN (3, 4). Despite their homology and shared preference for oligo(A) tails, TOE1 and PARN show different specificities for small RNA substrates (3–5). How do these two enzymes achieve specificity toward different substrates? Our findings suggest that this may be explained for a subset of target RNAs by their cap structures. While we find here that TOE1 recognizes the TMG cap characteristic of Pol II snRNAs, PARN was previously shown to be activated by the m7G cap (55–57), a 5′ modification found on a subset of small ncRNAs targeted by PARN, including TERC and a subset of snoRNAs. There are reports that TERC can also be observed with a TMG cap (62) and serve as a substrate for TOE1 (19), although we have been unable to detect processing of TERC by TOE1 in HEK293T cells (3). While cap structures may explain the differential activity of TOE1 and PARN toward a subset of substrates, how PARN achieves specificity for other substrates, including a majority of snoRNAs that are processed from introns and therefore remain uncapped (63), remains unresolved. A contributing factor may well be subcellular localization, with TOE1 known to co-localize with snRNAs in Cajal Bodies and PARN with snoRNAs and other substrates in the nucleolus. Similarly, the molecular basis for target preferences of other maturation exonucleases PNLDC1 and USB1 remains an important question for future study.

The 3′ ends of newly transcribed small non-coding RNAs serve as quality control hubs for competing exonucleases to drive 3′ end maturation or degradation. In the case of snRNAs, previous studies have shown that USB1 processes the Pol-III transcribed U6 and U6atac snRNAs leading to their stabilization (23, 64). For Pol II snRNAs, we previously identified TOE1 and the nuclear exosome as competing exonucleases acting on their 3′ ends to distinguish canonical snRNAs from unstable snRNA variants (5). Our findings here, that TOE1 is activated by late snRNA biogenesis features, Sm complex assembly, and cap trimethylation, explain how TOE1 can achieve specificity toward canonical snRNPs undergoing correct assembly. Unstable snRNA variants (5, 61) and canonical snRNAs experiencing defects in snRNP assembly (12, 50), likely undergo degradation because they fail to reach this late stage of snRNP biogenesis. This may represent a general principle in RNA quality control whereby substrate specificity relies on maturation enzymes that specifically recognize canonical RNAs undergoing proper biogenesis in competition with more promiscuous degradation enzymes to distinguish normal from aberrant RNAs and ultimately dictate their fate.

## Materials and Methods

### Cell Culture, RNA Interference, and Plasmid Transfections.

All cells were maintained in Dulbecco’s Modified Eagle Medium (DMEM, Gibco) supplemented with 10% Fetal Bovine Serum (FBS, Gibco) and 1% penicillin/streptomycin (Gibco) at 37 °C, 5% CO_2_. Mycoplasma testing was routinely performed. TOE1 was depleted in HEK 293 T-REx-derived TOE1-degron cells (5) by incubation with 600 μM auxin hormone Indole-3-Acetic Acid (IAA, Sigma) for 8 h. RNA interference was performed in HEK 293 T Flp-In cells (FIRT, Thermo Fisher) with 20 nM small interfering (si)RNA targeting genes of interest, or luciferase as a control (*SI Appendix*, Table S1), using siLentFect (Bio-Rad) transfection reagent according to the manufacturer’s recommendations at 72 and 24 h before cell harvest. Plasmid transfections were performed using 2 μg plasmid per 3.5-cm well plates using Lipofectamine 2000 (Life Technologies) transfection reagent according to the manufacturer’s recommendations at 48 h before harvest, unless specified otherwise.

### Global 3’ End Sequencing of Newly Transcribed Small RNAs.

HEK 293 T-REx TOE1-degron cells were treated to deplete TOE1 as described above. A total of 1.2 × 10^6^ TOE1-depleted, or control non-depleted, cells were incubated with 0.2 mM Ethynyl Uridine (EU; Thermo Fisher) for 8 h and harvested in 1 mL TRIzol reagent (Thermo Fisher). Total RNA was isolated according to the manufacturer’s recommendation. Small RNA from 35 μg of total RNA from each sample was isolated by separation in a 9% polyacrylamide/6 M urea denaturing gel. After Sybr Gold staining (Thermo Fisher), small RNAs between 100 and 500 nucleotides in length were cut out of the gel and eluted into 400 μL gel elution buffer (0.3 M sodium acetate pH 5.3, 1 mM EDTA, 0.1% SDS) by end-over-end rotation overnight at 4 °C. Eluted small RNAs were subsequently subjected to phenol:chloroform:isoamyl alcohol extraction followed by ethanol precipitation as previously described (3). Genomic DNA was removed using the Turbo DNA-free kit (Thermo Fisher) and ribosomal (r)RNAs were depleted using the RiboCOP rRNA depletion kit (Lexogen) per the manufacturer’s recommendations. The rRNA-depleted small RNA samples were subsequently subjected to FastAP/PNK treatment to remove RNA 5′ and 3′ phosphates by incubating samples with 2.5 μL 10× FastAP Buffer (Thermo Fisher), 0.5 μL RNaseOUT (Thermo Fisher), and 2.5 μL FastAP phosphatase (Thermo Fisher) in 25 μL total volume at 37 °C for 30 min, followed by supplementation with 10 μL 10× PNK buffer (NEB), 1 μL 0.1 M DTT, 1 μL Turbo DNase (2 U/µL; Thermo Fisher), 1 μL RNaseOUT (40 U/µL; Thermo Fisher), and 7 μL Polynucleotide Kinase (10 U/µL; NEB) in a total of 100 μL and incubation for 20 min at 37 °C. RNA samples were purified using an RNA Clean & Concentrator kit (Zymo Research). AG10N or AG11N RNA adaptors (10 µM; *SI Appendix*, Table S1) were ligated to the RNA 3′ ends of each sample by incubation at 25 °C for 75 min in a 20 µL total volume containing 1.8 μL DMSO (Sigma), 2 μL 10× T4 ligase buffer (NEB), 0.2 μL 0.1 M ATP (Thermo Fisher), 0.2 μL RNaseOUT (40 U/µL; Thermo Fisher), 8 μL 50% PEG 8000 (NEB), 1.3 μL T4 RNA ligase (30 U/µL; NEB). RNA samples were then subjected to extraction of newly transcribed RNAs using the Click-it nascent RNA capture kit (Thermo Fisher) as previously described (5). Reverse transcription was performed on the RNA capture beads in a total of 20 µL with 0.5 μL 20 μM AR17 primer (*SI Appendix*, Table S1), 2 μL 10× AffinityScript buffer (Agilent), 2 μL 0.1 M DTT, 0.8 μL 100 mM dNTPs, 0.3 μL RNaseOUT (40 U/µL; Thermo Fisher), 0.9 μL AffinityScript Reverse Transcriptase (Agilent) at 55 °C for 45 min, followed by 15 min incubation at 70 °C and 5 min of incubation at 85 °C to release cDNA. Excess primers and RNA was removed from the first-strand cDNA by incubating with 3.5 μL ExoSAP-IT (Thermo Fisher) at 37 °C for 15 min followed by addition of 3 μL of 1 M NaOH at 70 °C for 12 min. Then, 3 μL of 1 M HCl was added to the sample after the clean-up to adjust pH. A 3Tr3 adaptor (*SI Appendix*, Table S1) was ligated to cDNA 5′ ends by adding 0.8 μL of the adaptor at 80 µM to the cDNA sample along with 1 μL 100% DMSO (Sigma), 2 μL 10×T4 ligase buffer (NEB), 0.2 μL 0.1 M ATP (Thermo Fisher), 1.5 μL T4 RNA ligase (30 U/µL; NEB), and 1.1 μL double distilled water, and incubating the reaction at 25 °C for 16 h. The cDNA was amplified in two stages of PCR using Q5 DNA polymerase (NEB). For the first PCR reaction, the cDNA library was amplified using 3′ adaptor primer (AR17) and a primer complementary to the 5′ adaptor (RC_3Tr3) (*SI Appendix*, Table S1) for 8 cycles. The PCR product was purified by AMPure XP beads (Beckman Coulter) per the manufacturer’s recommendation. The second PCR reaction was performed using Illumina Trueseq D50X and D70X primers (*SI Appendix*, Table S1) for 18 cycles. Amplified cDNA was subsequently isolated by separation in a 3% agarose gel and isolation of cDNAs 200 to 600 base pairs in length (corresponding to 100 to 500 nucleotide RNAs plus adaptors) using the QIAquick gel extraction kit (Qiagen) according to the manufacturer’s recommendation. The library quality was monitored by qPCR for select genes and Tapestation (Agilent) analyses. In addition, 100-bp paired-end sequencing was performed on an Illumina NovaSeq 6000 platform per the manufacturer’s recommendation.

### Sequencing Data Analyses.

FASTQ files were first subjected to 3′ adaptor and PCR duplicate removal using custom python scripts (https://pypi.org/project/jla-demultiplexer/). For highly abundant small RNAs (U1, U2, U3, 7SL, and 7SK), 100,000 reads were selected for subsequent analyses by averaging three random samplings using seqtk (https://github.com/lh3/seqtk). This was done to prevent undercounting of these RNAs since their abundance in the full library exceeded the depth of the randomer used for detecting PCR duplicates (*SI Appendix*, Fig. S1*b*). For all other RNAs, the entire library was used. Reads were mapped to the human genome (version hg38) using STAR 2.7.8a (65). To diminish mis-mapping of canonical small RNA reads to small RNA variant genes, a three-pass alignment was applied. Briefly, reads were first mapped to the human hg38 genome (STAR --outFilterMultimapNmax 1000 --alignIntronMin 9999999 --outFilterMultimapScoreRange --outFilterMismatchNoverLmax 0.2) and reads mapping to small RNA genes were extracted using bedtools (66) and samtools (67). The mapped small RNA reads were then aligned to a custom FASTA database of canonical small RNA genes, each including 50 base pair upstream and downstream sequences (STAR --outFilterMultimapNmax 1000 --outFilterMultimapScoreRange 0 --outFilterMismatchNoverLmax 0.2 --outFilterMismatchNoverReadLmax 0.05 --clip5pNbases 20 0 --clip3pNbases 0 20 --alignIntronMin 9999999 --alignMatesGapMax 500 --alignEndsType EndToEnd --outReadsUnmapped Fastx). Reads that mapped to the canonical small RNA gene database were subsequently re-aligned to the full human h38 genome (STAR --outFilterMultimapNmax 1000 --outFilterMultimapScoreRange 0 --outFilterMismatchNoverLmax 0.025 --alignIntronMin 9999999 --alignMatesGapMax 500 --alignEndsType Local) and those that again mapped to canonical small RNA genes were extracted with bedtools. This step was performed to remove canonical small RNA reads with sequencing errors that may misalign with small RNA variant genes. Reads that failed to map to the canonical small RNA gene database were also re-aligned with the full human hg38 genome using the same settings and combined with the reads mapping to canonical small RNA genes. Gene-specific 3′ end information and graphs were subsequently generated using Tailer (68) (https://github.com/TimNicholsonShaw/tailer) using the global alignment mode.

### RNA Gene-Specific 3’ End Sequencing.

RNA was isolated using TRIzol (Thermo Fisher) per the manufacturer’s recommendation. RNA was subsequently treated with DNase I (1 U/µL; Zymo research) in DNase buffer (10 mM Tris-HCl pH 7.5, 2.5 mM MgCl_2_, 0.5 mM CaCl_2_) at 25 °C for 15 min, followed by extraction with phenol:chloroform:isoamyl alcohol and ethanol precipitation as previously described (5). For sequencing of newly transcribed RNAs, nascent RNA was captured as previously described (5). Gene-specific RNA 3′ end sequencing libraries were generated using gene-specific primers (*SI Appendix*, Table S1) and sequenced on an Illumina MiSeq platform as previously described (5).

### Isolation of Flag-Tagged TOE1.

HEK 293 T Flp-In cells (FIRT) cells were transfected with pcDNA5-Flag-TOE1 plasmid (13) as described above. First, 1 μg/mL of tetracycline was added 24 h before harvest to induce Flag-TOE1 expression. Then, 2 × 10^7^ Flag-TOE1-induced cells were lysed in 2.5 mL of isotonic lysis buffer (10 mM Tris-HCl pH 7.5, 150 mM NaCl, 2 mM EDTA, 0.1% Triton-X100, 1 mM PMSF, 2 µg/mL Aprotinin, 2 µg/mL Leupeptin) with 125 μg/mL RNase A. The lysate concentration was measured using BCA protein assay (Thermo Fisher) following the manufacturer’s protocol. Then, 40 µL of 50% anti-FLAG M2 agarose slurry (Sigma) was washed twice with 500 μL NET-2 (10 mM Tris HCl pH 7.4, 150 mM NaCl, 0.1% Triton-X100) before incubation with 1 to 5 mg of cell lysate with end-to-end rotation for 2 h at 4 °C. Beads were subsequently washed with 500 µL NET-2 eight times. Flag-TOE1 was eluted with 100 µL of NET-2 containing 150 ng/µL FLAG peptide (ApexBio) and 10% glycerol by rotating at 4 °C for 30 min. The isolated protein was detected by silver staining (Thermo Fisher) per the manufacturer’s recommendation. Eluates were aliquoted and stored at −80 °C until use.

### In Vitro snRNP Pull-Down and 3’ End Processing Assay.

HEK293T-Rex TOE1-degron cells were treated to deplete TOE1 as described above and co-transfected with 0.2 μg U1-WT and 2 μg Sm-mutant (*SI Appendix*, Table S2) expression plasmids as described above, 16 h prior to cell harvest. A total of 2 × 10^7^ cells were suspended in 0.2 mL of low salt lysis buffer (10 mM Tris-HCl pH 7.5, 60 mM NaCl, 2 mM EDTA, 0.1% Triton-X100, 1 mM PMSF, 2 µg/mL Aprotinin, 2 µg/mL Leupeptin, 0.1 µg/mL yeast total RNA, and 2 U/mL RNaseOUT) and incubated on ice for 10 min. Subsequently, 20 µL of 0.5 µM 2′OMe-RNA-oligo probe (*SI Appendix*, Table S1), 12 µL streptavidin magnetic beads (NEB), and 150 µL wash/binding buffer (0.5 M NaCl, 20 mM Tris-HCl pH 7.5, 1 mM EDTA) were incubated on ice for 30 min with occasional agitation. Then, 200 to 400 µL of cell lysate was added to each tube and incubated at 4 °C for 2 h. Beads were washed twice with 100 µL wash/binding buffer followed by one wash with 100 µL low salt wash buffer (20 mM Tris-HCl pH 7.5, 150 mM NaCl, 1 mM EDTA), and five times with 100 µL EDTA-free low salt buffer (20 mM Tris-HCl pH 7.5, 150 mM NaCl, 0.1% NP-40). After washes, 200 µL of reaction buffer (20 mM HEPES pH 7.4, 2 mM MgCl_2_, 0.1 mg/mL bovine serum albumin, 1 mM spermidine, 0.1% NP-40) with 0.5 U/μL RNaseOUT (Invitrogen), 0.5 μg/μL yeast total RNA and 0 to 100 nM of Flag-TOE1 was added to the isolated snRNPs. Reaction tubes were incubated with gentle rotation at 30 °C for 30 min followed by incubation at 4 °C for 30 min. The beads were then isolated using a magnetic stand and resuspended in 100 µL reaction buffer with 100 µL formamide/2 mM EDTA and incubated at 80 °C for 5 min. Then, 1 mL of TRIzol (Thermo Fisher) was added to each sample. Two biological repeats were obtained using individual snRNP pull-down and two individual Flag-TOE1 preparations. RNA extraction, 3′ end sequencing library preparation, sequencing, and data analysis were performed as described above.

### In Vitro Transcription and 3’ End Processing Assay.

TMG-, m7G-capped, or uncapped U1 snRNAs were produced by in vitro transcription using T7 RNA polymerase (NEB). Briefly, 500 ng of DNA template, produced by PCR from a U1 snRNA expression plasmid (50) using T7_U1-Forward primer with T7_U1-Reverse_20geno or T7_U1-Reverse-10A primers (*SI Appendix*, Table S1), was mixed with 8 mM m7G cap analog (ARCA; NEB), TMG cap analog (Jena Bioscience) or no cap analog, 0.05 M DTT, 1 μL RNaseOUT (2 U/µL; Thermo Fisher), 2 μL NTP buffer mix (1.34 mM final concentration for each NTP, NEB), 2 μL T7 RNA Polymerase Mix (NEB) in a total of 20 μL. In vitro transcription was performed at 37 °C for 16 h. RNA products were purified using an RNA Clean & Concentrator kit (Zymo Research) and quantified using a Nanodrop (Thermo Fisher) per the manufacturer's recommendations. For TOE1 3′ end processing assays, 10 ng of RNA was mixed with 100 nM Flag-tagged or 50 nM His-tagged TOE1 in 10 μL reaction buffer containing 20 mM HEPES pH 7.4, 2 mM MgCl_2_, 100 μg/mL BSA, 1 mM Spermidine, 0.1% NP-40, 0.5 U/μL RNaseOUT, 0.5 μg/μL yeast total RNA. The processing reaction was performed at 37 °C for 0 to 120 min. Reactions were terminated with 10 µL of denaturing load buffer (95% formamide, 10 mM EDTA, 0.01% Bromophenol Blue, 0.01% Xylene Cyanol) followed by incubation at 80 °C for 10 min to denature the RNA. The reaction products were subsequently separated in a 7% acrylamide/6 M urea denaturing gel. RNA was stained with Sybr Gold (Thermo Fisher) by nutation in the dark for 30 min. RNA was imaged by a Typhoon gel imager (Amersham). Gel images were analyzed using ImageJ. The processing reactions were repeated twice using the same recombinant TOE1 preparations.

### qPCR assays.

AR17 (*SI Appendix*, Table S1)-primed cDNA was amplified using Fast SYBR Green master mix (Thermo Fisher) with primers for RNAs of interest (*SI Appendix*, Table S1) on a QuantStudio Real-Time PCR system (Thermo Fisher). Relative levels were quantified using the ΔΔC_t_ method (69).

### Western Blotting.

Western blots were performed by separating proteins in SDS-polyacrylamide gels followed by transfer to nitrocellulose membranes using standard procedures. Membranes were incubated overnight at 4 °C with rabbit polyclonal anti-CAF1Z/TOE1 (13) at 1:1000, rabbit polyclonal anti-UPF1 (70) at 1:1000, mouse monoclonal anti-SNRPB (Thermo Fisher) at 1:500, each in PBS with 0.1% Tween (PBST) and 5% nonfat milk, or with mouse monoclonal anti-U1-70 K (Synaptic System) at 1:1000 in PBST with 5% BSA. Secondary antibodies were goat anti-rabbit IRDye 680RD (LI-COR) at 1:15000, HPR donkey anti-rabbit IgG (H + L) (Thermo Fisher), or HPR goat anti-mouse rabbit IgG (H + L) (Thermo Fisher) at 1:20000 in PBST with 5% nonfat milk. Western blots were visualized using an Odyssey Fc imaging system (LI-COR).

## Supplementary Material

Appendix 01 (PDF)

## Data Availability

RNA sequencing data have been deposited to the Gene Expression Omnibus under Accession No. GSE240774 (51).
