# Supplementary material for: Sm complex assembly and 5′ cap trimethylation promote selective processing of snRNAs by the 3′ exonuclease TOE1
Source: Proc Natl Acad Sci U S A. 2024 Jan 9;121(3):e2315259121. doi: 10.1073/pnas.2315259121 (PMC10801842; doi:10.1073/pnas.2315259121)
Supplement: Supplementary file 1 — Appendix 01 (PDF) [file pnas.2315259121.sapp.pdf]

PNAS

Supporting Information for

Sm complex assembly and 5’ cap trimethylation promote selective processing of snRNAs by the 3' exonuclease TOE1

Tiantai Ma, Erica S. Xiong, Rea M. Lardelli*, and Jens Lykke-Andersen*

*Jens Lykke-Andersen, Rea M. Lardelli

Email: jlykkeandersen@ucsd.edu, reamartine@gmail.com

This PDF file includes:

Supplementary text
Supplementary Figures S1-S7
Supplementary Tables S1-S2

Supplementary Figures

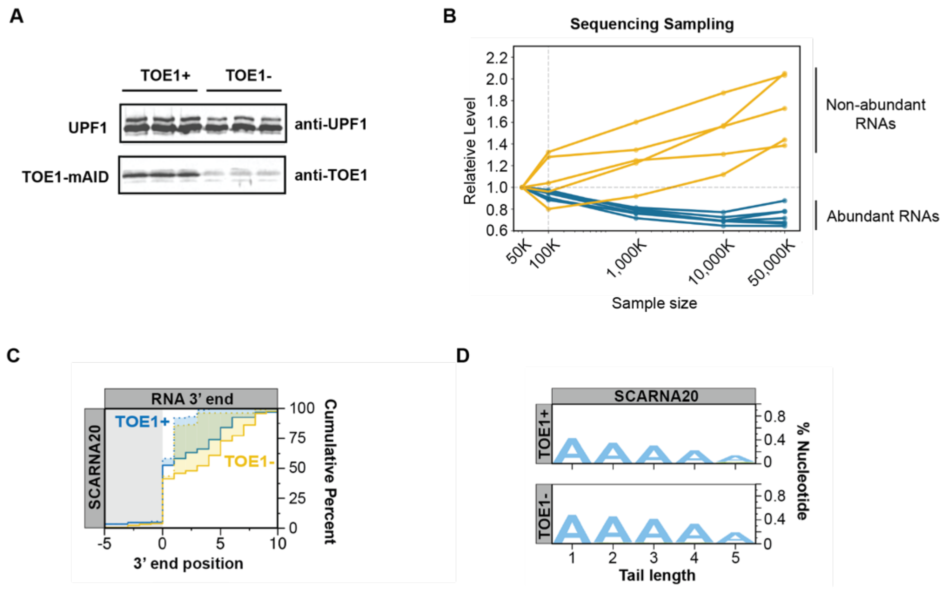

Supplementary Figure S1 related to Figure 1. TOE1 shows specificity towards Pol II snRNAs. (A) Western blots showing depletion of TOE1. UPF1 serves as a loading control. **(B)** Plot showing the fraction of non-duplicate mapped reads for abundant small ncRNAs (RNU1, RNU2, RNU2-2P, SNORD3, RN7SL1, RN7SL2, RN7SL3, RN7SK) compared with select low abundance RNAs (SNORD13, SNORA71A, SNORA70, SNORA77B, SNORA71C) following sampling of 50k to 50,000k total reads, as normalized to sampling of 50k total reads. The vertical dashed line shows the sampling size (100k reads) used for analyses of the shown abundant small ncRNAs. All other RNAs were analyzed from the full library. **(C)** Cumulative plot showing distributions of 3' end positions for SCARNA20 in control (TOE1+, blue) and TOE1-depleted (TOE1-, yellow) conditions. **(D)** Sequence logo plots representing the percentage of post-transcriptionally added nucleotides for SCARNA20 under control (TOE1+) or TOE1-depleted (TOE1-) conditions.

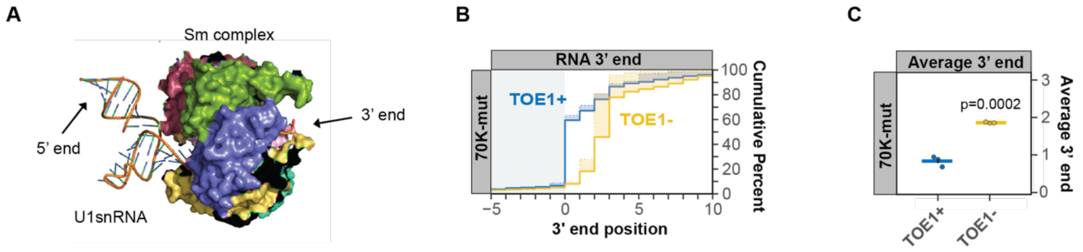

Supplementary Figure S2 related to Figure 2. Mutations in Sm complex- and U1-70K-binding motifs of U1 snRNA inhibit 3' end processing. (A) Structure of U1 snRNA associated with the Sm complex based on X-ray crystallography (69). **(B)** Cumulative plot showing 3' end distributions for the 70K-mutant U1 snRNA under TOE1+ (blue) or TOE1- (yellow) conditions. **(C)** Average 3' end positions for the 70K mutant U1 snRNA in TOE1+ (blue) and TOE1- (yellow) conditions.

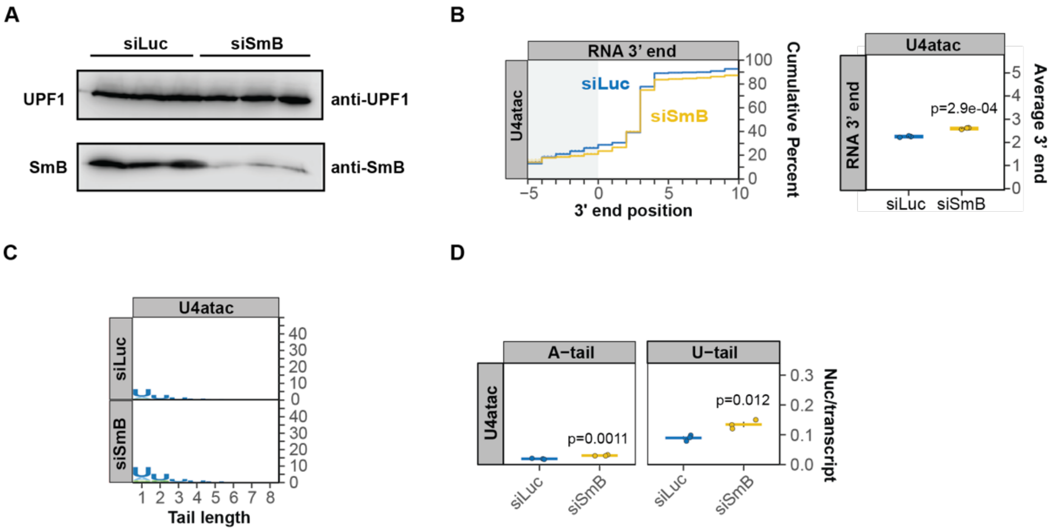

Supplementary Figure S3 related to Figure 3. Sm complex depletion impairs 3' end processing of multiple Pol II snRNAs. (A) Western blot showing depletion of SmB. UPF1 serves as a loading control. **(B)** Left, cumulative plots showing 3' end distributions of nascent minor class Pol II snRNA U4atac in control (siLuc; blue) versus SmB (siSmB; yellow) depleted conditions. The average of three individual biological repeats is plotted. Right, average 3' end lengths for U4atac snRNA. Dots represent individual biological repeats and SEMs are shown as vertical black lines. P-values were calculated by Student's two-tailed t-test. **(C)** Sequence logo plots representing the percentage of post-transcriptionally added nucleotides for U4atac snRNA under control (siLuc) or SmB knock down (siSmB) conditions. **(D)** Average number of post-transcriptionally added adenosine or uridine nucleotides per transcript for U4atac snRNA, plotted as in panel *B*.

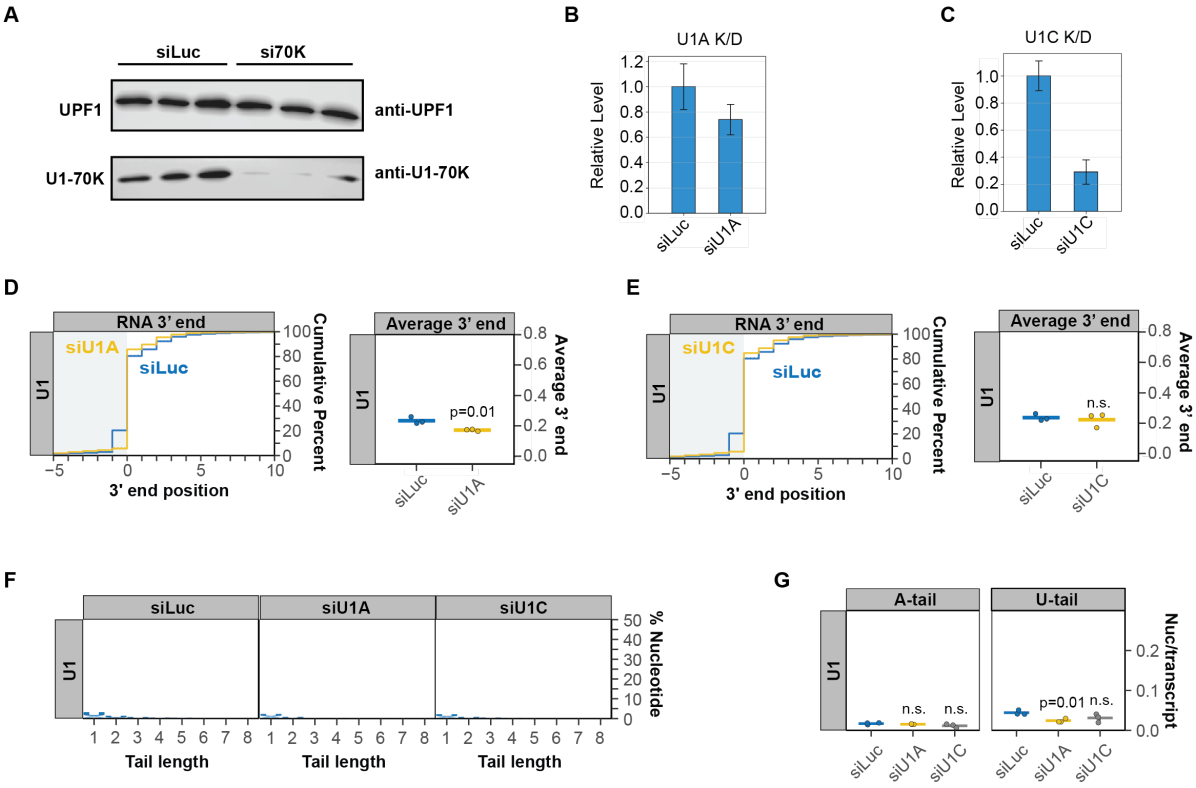

Supplementary Figure S4 related to Figure 4. U1-70K compensates for a suboptimal Sm binding motif to stimulate U1 snRNA 3' end processing. (A) Western blot showing depletion of U1-70K. UPF1 serves as a loading control. **(B, C)** Relative levels of U1A (panel *B*) and U1C (panel *C*) mRNAs in U1A- and U1C-depleted conditions, respectively, compared to control (siLuc) conditions, measured by RT-qPCR. Samples were normalized to the average level of GAPDH and Mitochondrial 12S rRNA. Error bars represent SEMs from three individual experiments. **(D, E)** Left, cumulative plots showing 3' end distributions of nascent U1 snRNA in control (siLuc; blue) versus U1A (panel *D*) or U1C (panel *E*) (yellow) knock-down conditions. Right, average 3' end lengths for U1 snRNA. Dots represent individual biological repeats and SEMs are shown as vertical black lines. P-values were calculated by Student's two-tailed t-test. **(F)** Sequence logo plots representing the percentage of post-transcriptionally added nucleotides for nascent U1 snRNA under control (siLuc), U1A knock-down (siU1A), and U1C knock-down (siU1C) conditions. **(G)** Average number of post-transcriptionally added adenosine or uridine nucleotides per transcript, plotted as in panels *D* and *E*.

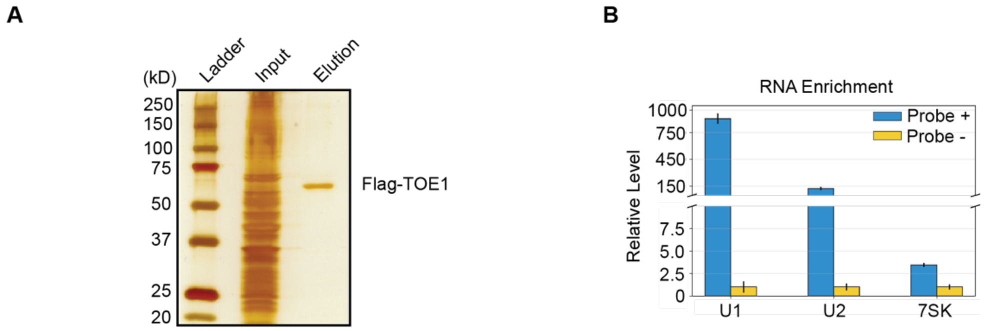

Supplementary Figure S5 related to Figure 6. TOE1 directly recognizes the Sm complex-assembled U1 snRNP. (A) Representative silver-stained gel showing the Flag-TOE1 IP input and elution fractions. kD = kilodaltons. **(B)** Bar plots showing the relative enrichment of U1, U2, and 7SK RNAs following 2'-OMe-RNA oligonucleotide pull-down, measured by RT-qPCR, in pull-down (probe+, Blue) and negative control (probe-, yellow) conditions. Mitochondrial 12S rRNA served as an internal control.

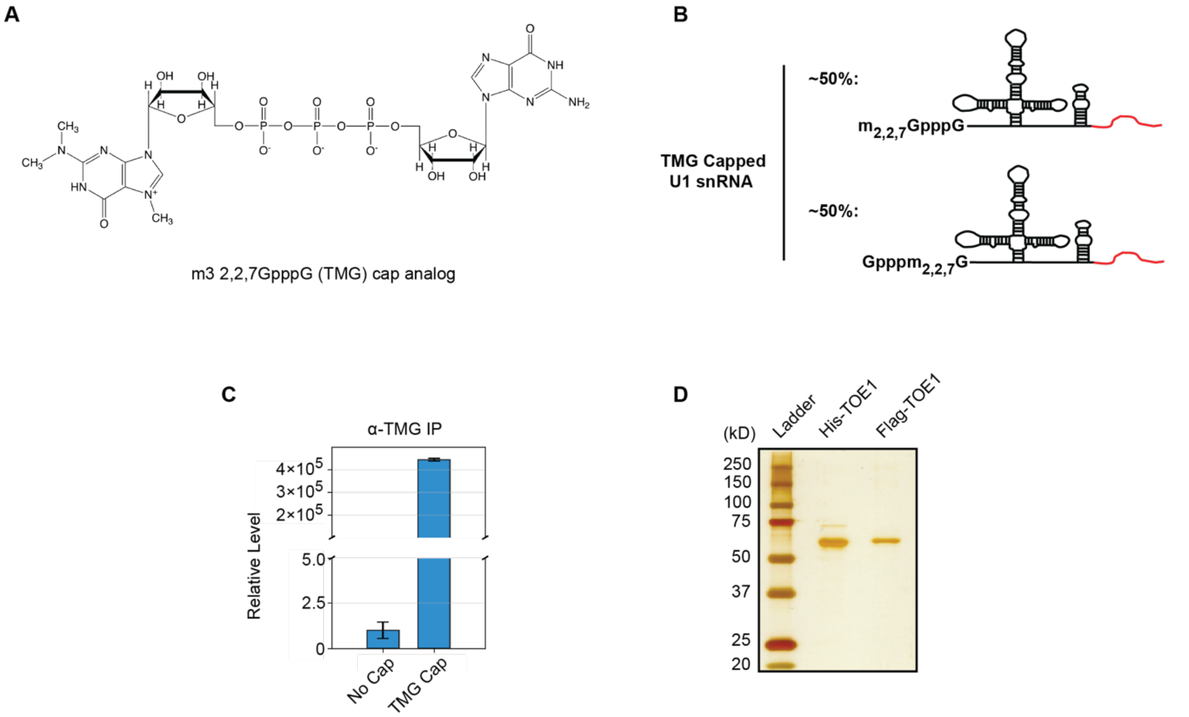

Supplementary Figure S6 related to Figure 7. snRNA processing by TOE1 is stimulated by the 5' TMG cap. (A) Structure of the TMG cap analog. **(B)** The TMG-capped U1 snRNA is a mixture of $m_{2,2,7}$GpppG-capped and Gpppm$_{2,2,7}$G-capped U1 snRNA. **(C)** Relative level of *in vitro* transcribed U1 snRNA with TMG cap or no cap following anti-TMG Immunoprecipitation, measured by RT-qPCR. Samples were normalized to input. **(D)** Representative silver-stained gel showing purified His- and Flag-tagged TOE1 proteins.

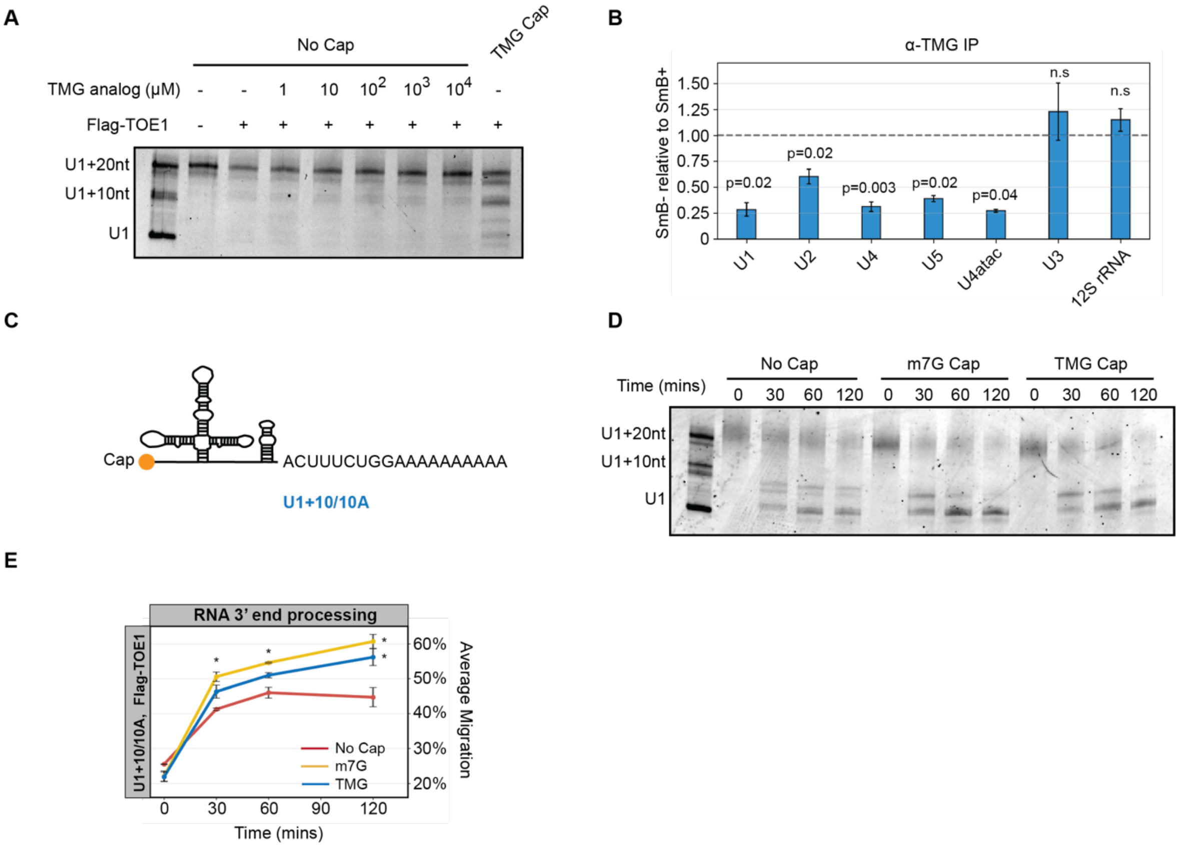

Supplementary Figure S7. (A) Representative denaturing gel showing U1+20 uncapped RNA substrate incubated with TMG cap analog at indicated concentrations and Flag-TOE1 *in vitro* for one hour. **(B)** Relative levels of Pol II snRNAs, U3 snoRNA, and 12S rRNA following TMG-IP, measured by qRT-PCR, normalizing SmB knock down (SmB-) conditions to control (SmB+) conditions. P-values are calculated by Student's two-tailed t-test comparing SmB- to SmB+ conditions. **(C)** Schematic of *in vitro* transcribed U1 snRNA with a 10-nucleotide genomic-encoded tail followed by a 10-nucleotides oligo(A) tail (U1+10/10A). **(D, E)** Same as Figure 7 panels *B* and *C*, using U1+10/10A RNA as the substrate for Flag-TOE1 processing.

Supplementary TableS1

Gene-specific sequencing primers

U1-01	CAGACGTGTGCTCTTCCGATCT ATCG ATGATCACGAAGGTGGTTTT
U1-02	CAGACGTGTGCTCTTCCGATCT GAACG ATGATCACGAAGGTGGTTTT
U1-03	CAGACGTGTGCTCTTCCGATCT GTTGTA ATGATCACGAAGGTGGTTTT
U1-04	CAGACGTGTGCTCTTCCGATCT CTACCAT ATGATCACGAAGGTGGTTTT
U1-05	CAGACGTGTGCTCTTCCGATCT TGGCTTCA ATGATCACGAAGGTGGTTTT
U1-06	CAGACGTGTGCTCTTCCGATCT ACTACGTGT ATGATCACGAAGGTGGTTTT
U1-07	CAGACGTGTGCTCTTCCGATCT TCGTACACAG ATGATCACGAAGGTGGTTTT
U1-08	CAGACGTGTGCTCTTCCGATCT CACAGTATGAC ATGATCACGAAGGTGGTTTT
U1-09	CAGACGTGTGCTCTTCCGATCT ATGCTGACAAGT ATGATCACGAAGGTGGTTTT
U1-10	CAGACGTGTGCTCTTCCGATCT GTCAAGTGCTAGC ATGATCACGAAGGTGGTTTT
U1-11	CAGACGTGTGCTCTTCCGATCT CAATGTACGAACGT ATGATCACGAAGGTGGTTTT
U1-12	CAGACGTGTGCTCTTCCGATCT TAGC ATGATCACGAAGGTGGTTTT
U1-37	CAGACGTGTGCTCTTCCGATCT AATC TTACCTGGCAGGGGAGATA
U1-38	CAGACGTGTGCTCTTCCGATCT TTCGA TTACCTGGCAGGGGAGATA
U1-39	CAGACGTGTGCTCTTCCGATCT CCAAAA TTACCTGGCAGGGGAGATA
U1-40	CAGACGTGTGCTCTTCCGATCT GGTTTTG TTACCTGGCAGGGGAGATA
U2-01	CAGACGTGTGCTCTTCCGATCT TACG GGAGATGGAATAGGAGCTTGC
U2-02	CAGACGTGTGCTCTTCCGATCT CTGCT GGAGATGGAATAGGAGCTTGC
U2-03	CAGACGTGTGCTCTTCCGATCT CAATCA GGAGATGGAATAGGAGCTTGC
U2-04	CAGACGTGTGCTCTTCCGATCT GTAGATG GGAGATGGAATAGGAGCTTGC
U2-05	CAGACGTGTGCTCTTCCGATCT AGCCATAG GGAGATGGAATAGGAGCTTGC
U2-06	CAGACGTGTGCTCTTCCGATCT TGTACAGCA GGAGATGGAATAGGAGCTTGC
U3-01	CAGACGTGTGCTCTTCCGATCT TACA CTCTGAACGTGTAGAGCAC
U3-02	CAGACGTGTGCTCTTCCGATCT ATGTA CTCTGAACGTGTAGAGCAC
U3-03	CAGACGTGTGCTCTTCCGATCT GCACGA CTCTGAACGTGTAGAGCAC
U3-04	CAGACGTGTGCTCTTCCGATCT AGTGCTA CTCTGAACGTGTAGAGCAC
U3-05	CAGACGTGTGCTCTTCCGATCT CAACTCGA CTCTGAACGTGTAGAGCAC
U3-06	CAGACGTGTGCTCTTCCGATCT TCTTGGATA CTCTGAACGTGTAGAGCAC
U4-01	CAGACGTGTGCTCTTCCGATCT CGTA GCAGTATCGTAGCCAATGAGG
U4-02	CAGACGTGTGCTCTTCCGATCT CGTTC GCAGTATCGTAGCCAATGAGG
U4-03	CAGACGTGTGCTCTTCCGATCT TACAAC GCAGTATCGTAGCCAATGAGG
U4-04	CAGACGTGTGCTCTTCCGATCT ATGGTAG GCAGTATCGTAGCCAATGAGG
U4-05	CAGACGTGTGCTCTTCCGATCT TGATGGCA GCAGTATCGTAGCCAATGAGG
U4-06	CAGACGTGTGCTCTTCCGATCT ACACGTTGT GCAGTATCGTAGCCAATGAGG
U5-04	CAGACGTGTGCTCTTCCGATCT GATGGTA ATACTCTGGTTTCTCTTCAGATCG
U5-05	CAGACGTGTGCTCTTCCGATCT ACCGAAGT ATACTCTGGTTTCTCTTCAGATCG
U5-06	CAGACGTGTGCTCTTCCGATCT TGATGCACA ATACTCTGGTTTCTCTTCAGATCG
U5-07	CAGACGTGTGCTCTTCCGATCT GACATTCGTA ATACTCTGGTTTCTCTTCAGATCG

U5-08	CAGACGTGTGCTCTTCCGATCT ATGGACTAGTA ATACTCTGGTTTCTCTTCAGATCG
U5-09	CAGACGTGTGCTCTTCCGATCT ATCATG ATACTCTGGTTTCTCTTCAGATCG
U5-10	CAGACGTGTGCTCTTCCGATCT TAGCATG ATACTCTGGTTTCTCTTCAGATCG
U4atac-01	CAGACGTGTGCTCTTCCGATCT CACT CATCCTTTTCTTGGGGTTGC
U4atac-02	CAGACGTGTGCTCTTCCGATCT TGTGC CATCCTTTTCTTGGGGTTGC
U4atac-03	CAGACGTGTGCTCTTCCGATCT ATTCCA CATCCTTTTCTTGGGGTTGC
U4atac-04	CAGACGTGTGCTCTTCCGATCT GAATTCA CATCCTTTTCTTGGGGTTGC
U4atac-05	CAGACGTGTGCTCTTCCGATCT GGTTAACC CATCCTTTTCTTGGGGTTGC
U4atac-06	CAGACGTGTGCTCTTCCGATCT TACCATTGA CATCCTTTTCTTGGGGTTGC

qPCR primers

U1-forward	GCACTCCGGATGTGCTGACCC
U1-reverse	CAGGGGAAAGCGCGAACGCAG
U2-forward	TTTGGCTAAGATCAAGTGTAGTATCTG
U2-reverse	AATCCATTTAATATATTGTCCTCGGAT
U3-forward	AGAGGTAGCGTTTTCTCCTGAGCG
U3-reverse	ACCACTCAGACCGCGTTCTC
U4-forword	GCGCGATTATTGCTAATTGAAA
U4-reverse	AAAAATTGCCAATGCCGACTA
U5-forward	GGTTTCTCTTCAGATGGCATAAATC
U5-reverse	CTCAAAAAATTGGGTTAAGACTCAGA
U4atac-forward	CCATCCTTTTCTTGGGGTTG
U4atac-reverse	TAGTTGATGCGGGTGTGTTG
7sk-forward	GAGGGCGATCTGGCTGCGACAT
7sk-reverse	ACATGGAGCGGTGAGGGAGGAA
12s-forward	ATGCAGCTCAAAACGCTTAGC
12s-reverse	GCTGGCACGAAATTGACCAA
GAPDH-forward	ACAACTTTGGTATCGTGGAAGG
GAPDH-reverse	GCCATCACGCCACAGTTTC
U1A-forward	GAAGAGGAAGCCCAAGAGCC
U1A-reverse	GGATTCTCAGAAAGAGGCTGGG
U1C-forward	ACTGCGATACATACCTCACCC
U1C-reverse	GTTGAAATGCAGCCGTTGTT

Cloning primers

T7_U1-Forward	TAATACGACTCACTATAGGGAGACCCAAGCTTATACTTACCTGGCAGG
T7_U1-Reverse-20geno	CTTTTGAAACTCCAGAAAGTCAG
T7_U1-Reverse-10A	TTTTTTTTTTCCAGAAAGTCAGGGGAA

RNA adapter

AG-10N	/5Phos/AGNNNNNNNNNNAGAUCGGAAGAGCGUCGUG/3SpC3/
AG-11N	/5Phos/AGNNNNNNNNNNNAGAUCGGAAGAGCGUCGUG/3SpC3/

5' cDNA adapter

3Tr3	/5Phos/AGATCGGAAGAGCACACGTCTG/3SpC3/

library prep and sequencing

AR-17	ACACGACGCTCTTCCGA
RC_3Tr3	CAGACGTGTGCTCTTCCGATCT
PCR_F_D501	AATGATACGGCGACCACCGAGATCTACAC TATAGCCT ACACTCTTTCCCTACACGACGCTCTTCCGATCT
PCR_F_D502	AATGATACGGCGACCACCGAGATCTACAC ATAGAGGC ACACTCTTTCCCTACACGACGCTCTTCCGATCT
PCR_F_D503	AATGATACGGCGACCACCGAGATCTACAC CCTATCCT ACACTCTTTCCCTACACGACGCTCTTCCGATCT
PCR_F_D701	CAAGCAGAAGACGGCATA CGAGAT CGAGTAAT GTGACTGGAGTTCAGACGTGTGCTCTTCCGATC
PCR_F_D702	CAAGCAGAAGACGGCATA CGAGAT TCTCCGGA GTGACTGGAGTTCAGACGTGTGCTCTTCCGATC
PCR_F_D703	CAAGCAGAAGACGGCATA CGAGAT AATGAGCGGTGACTGGAGTTCAGACGTGTGCTCTTCCGATC
PCR_F_D704	CAAGCAGAAGACGGCATA CGAGAT GGAATCTCGTGACTGGAGTTCAGACGTGTGCTCTTCCGATC
PCR_F_D705	CAAGCAGAAGACGGCATA CGAGAT TTCTGAATGTGACTGGAGTTCAGACGTGTGCTCTTCCGATC
PCR_F_D706	CAAGCAGAAGACGGCATA CGAGAT ACGAATTCGTGACTGGAGTTCAGACGTGTGCTCTTCCGATC

siRNAs

siControl (siLuciferase)	CGUACGCGGAAUACUUCGAUU
siSNRPB (SmB)	CCGUAAGGCUGUACAUAGUUU
siU1-70K	GGUCUACAGUAAGCGGUCAUU
siSNRPA (U1A)	ON-TARGETplus Human SNRPA (6626) siRNA - Individual, 2 nmol, J-019435-17-0002, Dharmacon
siSNRPC (U1C)	CGGAAUGACUCGACCAGACUU

2'OMe-RNA-oligo probe

5'- mAmGmGmUmAmAmGmUmAmUmAmAmGmGmU

mAmAmGmUmAmUmAmAmGmGmUmAmAmGmUmAmUmA/3Bio/ -3'

Supplementary TableS2

snRNA mutants

Bar-coded U1 WT	ATACTTACCTGGCAGGGGAGATACCATGATCACGAAGGTGGTTTTCCCAGGGCG
	AGGCTTATCCATTGCACTCCGGATGTGCTGACCCCTGCGATTTCCCCAAAGCTG
	GGAAACTCGACTGCATAATTTGTGGTAGTGGGGGACTGCGTTCGCGCTTTCCCCTG
Bar-coded Sm-mut	ATACTTACCTGGCAGGGGAGATACCATGATCACGAAGGTGGTTTTCCCAGGGCG
	AGGCTTATCCATTGCACTCCGGATGTGCTGACCCCTGCGATTTCCCCAAAGCTG
	GGAAACTCGACTGCATAATCCCCGGTAGTGGGGGACTGCGTTCGCGCTTTCCCCTG
Bar-coded 70K-mut	ATACTTACCTGGCAGGGGAGATACCAGGCCCACGAAGGTGGTTTTCCCAGGGCG
	AGGCTTATCCATTGCACTCCGGATGTGCTGACCCCTGCGATTTCCCCAAAGCTG
	GGAAACTCGACTGCATAATTTGTGGTAGTGGGGGACTGCGTTCGCGCTTTCCCCTG
Bar-coded U1A-mut	ATACTTACCTGGCAGGGGAGATACCATGATCACGAAGGTGGTTTTCCCAGGGCG
	AGGCTTATCCCTTCCAATCCGGATGTGCTGACCCCTGCGATTTCCCCAAAGCTG
	GGAAACTCGACTGCATAATTTGTGGTAGTGGGGGACTGCGTTCGCGCTTTCCCCTG
Bar-coded SuperU1-mut	ATACTTACCTGGCAGGGGAGATACCATGATCACGAAGGTGGTTTTCCCAGGGCG
	AGGCTTATCCATTGCACTCCGGATGTGCTGACCCCTGCGATTTCCCCAAAGCTG
	GGAAACTCGACTGCATAATTTTTGGTAGTGGGGGACTGCGTTCGCGCTTTCCCCTG
Bar-coded SuperU1+70K-mut	ATACTTACCTGGCAGGGGAGATACCAGGCCCACGAAGGTGGTTTTCCCAGGGCG
	AGGCTTATCCATTGCACTCCGGATGTGCTGACCCCTGCGATTTCCCCAAAGCTG
	GGAAACTCGACTGCATAATTTTTGGTAGTGGGGGACTGCGTTCGCGCTTTCCCCTG
Bar-coded U1v15	ATACTTACTTGGCGGGGGAGATACCATGATCACGAAGGTGGTTTTCTCAGGGCG
	AGGCTTATCCGTTATGTTCCGGGTGTACTGACCCCTGCCATTTTCCCCCAAGCTG
	AGGAACTCGACTGCATAACTTGTGATAGTAGGGGACTGCGTTCGCGCTTTCCCCTG
Bar-coded v15-U1A	ATACTTACCTGGCAGGGGAGATACCATGATCACGAAGGTGGTTTTCCCAGGGCG
	AGGCTTATCCGTTATGTTCCGGATGTGCTGACCCCTGCGATTTCCCCAAAGCTG
	GGAAACTCGACTGCATAATTTGTGGTAGTGGGGGACTGCGTTCGCGCTTTCCCCTG
Bar-coded v15-Sm	ATACTTACCTGGCAGGGGAGATACCATGATCACGAAGGTGGTTTTCCCAGGGCG
	AGGCTTATCCATTGCACTCCGGATGTGCTGACCCCTGCGATTTCCCCAAAGCTG
	GGAAACTCGACTGCATAACTTGTGATAGTGGGGGACTGCGTTCGCGCTTTCCCCTG
Bar-coded v15-SmWT	ATACTTACTTGGCGGGGGAGATACCATGATCACGAAGGTGGTTTTCTCAGGGCG
	AGGCTTATCCGTTATGTTCCGGGTGTACTGACCCCTGCCATTTTCCCCCAAGCTG

	AGGAACTCGACTGCATAATTTGTGGTAGTAGGGGACTGCGTTCGCGCTTTCCCCTG
Bar-coded v15-super	ATACTTACTTGGCGGGGGAGATACCATGATCACGAAGGTGGTTTTCTCAGGGCG<br>AGGCTTATCCGTTATGTTCCGGGTGTACTGACCCCTGCCATTTTCCCCCAAGCTG<br>AGGAACTCGACTGCATAATTTTTGGTAGTAGGGGACTGCGTTCGCGCTTTCCCCTG
